# Structure–Activity Relationship Studies on Novel Antiviral Agents for Norovirus Infections

**DOI:** 10.3390/microorganisms9091795

**Published:** 2021-08-24

**Authors:** Salvatore Ferla, Carmine Varricchio, William Knight, Pui Kei Ho, Fabiana Saporito, Beatrice Tropea, Giulio Fagan, Ben Matthew Flude, Federica Bevilacqua, Nanci Santos-Ferreira, Jana Van Dycke, Johan Neyts, Andrea Brancale, Joana Rocha-Pereira, Marcella Bassetto

**Affiliations:** 1 Medical School, Swansea University, Swansea SA28PP, UK; salvatore.ferla@swansea.ac.uk; 2 School of Pharmacy and Pharmaceutical Sciences, Cardiff University, Cardiff CF10 3NB, UK; VarricchioC@cardiff.ac.uk (C.V.); hopk@cardiff.ac.uk (P.K.H.); sap.fabi@gmail.com (F.S.); beatrice.tropea@gmail.com (B.T.); fagan.giulio@gmail.com (G.F.); federica1bevilacqua@gmail.com (F.B.); 3 Department of Chemistry, Faculty of Science and Engineering, Swansea University, Swansea SA28PP, UK; 952730@swansea.ac.uk (W.K.); 959957@Swansea.ac.uk (B.M.F.); 4 Rega Institute for Medical Research, University of Leuven, 3000 Leuven, Belgium; nanci.ferreira@kuleuven.be (N.S.-F.); jana.vandycke@kuleuven.be (J.V.D.); johan.neyts@kuleuven.be (J.N.); joana.rochapereira@kuleuven.be (J.R.-P.)

**Keywords:** antiviral agents, human norovirus, computer-aided drug design, SARs

## Abstract

Human norovirus is the leading cause of acute gastroenteritis worldwide, affecting every year 685 million people. Norovirus outbreaks are associated with very significant economic losses, with an estimated societal cost of 60 billion USD per year. Despite this, no therapeutic options or vaccines are currently available to treat or prevent this infection. An antiviral therapy that can be used as treatment and as a prophylactic measure in the case of outbreaks is urgently needed. We previously described the computer-aided design and synthesis of novel small-molecule agents able to inhibit the replication of human norovirus in cell-based systems. These compounds are non-nucleoside inhibitors of the viral polymerase and are characterized by a terminal *para*-substituted phenyl group connected to a central phenyl ring by an amide-thioamide linker, and a terminal thiophene ring. Here we describe new modifications of these scaffolds focused on exploring the role of the substituent at the *para* position of the terminal phenyl ring and on removing the thioamide portion of the amide-thioamide linker, to further explore structure-activity relationships (SARs) and improve antiviral properties. According to three to four-step synthetic routes, we prepared thirty novel compounds, which were then evaluated against the replication of both murine (MNV) and human (HuNoV) norovirus in cells. Derivatives in which the terminal phenyl group has been replaced by an unsubstituted benzoxazole or indole, and the thioamide component of the amide-thioamide linker has been removed, showed promising results in inhibiting HuNoV replication at low micromolar concentrations. Particularly, compound 28 was found to have an EC_50_ against HuNoV of 0.9 µM. Although the most active novel derivatives were also associated with an increased cytotoxicity in the human cell line, these compounds represent a very promising starting point for the development of new analogues with reduced cytotoxicity and improved selectivity indexes. In addition, the experimental biological data have been used to create an initial 3D quantitative structure-activity relationship model, which could be used to guide the future design of novel potential anti-norovirus agents.

## 1. Introduction

Previously known as the Norwalk Virus [1], human norovirus (HuNoV) is the main responsible for acute gastroenteritis outbreaks worldwide [2]. Norovirus infections represent a serious health concern especially in the developing countries, and they cause every year~214,000 deaths, particularly in immunocompromised patients, the elderly and children under the age of five [3]. Norovirus also represents a major financial burden, with $4.2 billion in direct health system costs and $60.3 billion in societal costs annually [4]. Currently, there are no vaccines or antiviral agents approved for this viral infection, with an urgent need for the development of therapeutic measures to be used for the treatment of infections or as a prophylactic strategy during outbreaks.

Noroviruses (NoV) belong to the *Caliciviridae* family, which have a linear, positive-sense, single-stranded RNA genome [5], and can be classed into 10 different genogroups (GI – GX) present in humans and animals, with GII, GIV, GVIII and GIX causing disease in humans [6]. Genogroups are further divided into genotypes, with genotype 4 of genogroup 2 (GII.4) being the predominant in outbreaks of acute gastroenteritis [7]. The HuNoV genome is organized into three open reading frames, which encode for seven non-structural proteins (NS1-7), the major structural proteins VP1 and VP2, and a minor structural protein [8]. On top of different vaccines currently under development [9], diverse antiviral strategies are being explored, including inhibition of attachment and entry, direct interference with viral non-structural proteins, and interference with host factors that contribute to the viral life cycle [10]. To date, only one antiviral agent has completed clinical trials, the broad-spectrum antimicrobial agent nitazoxanide [10], whose target for inhibition of norovirus infections is unknown, and whose efficacy in clinical trials remains unclear [10]. A second antiviral agent, CMX521, a nucleoside analogue targeting the viral polymerase, has entered Phase I clinical trials for the treatment of norovirus infections in 2018, but further updates on the efficacy of this molecule have yet to be released [11].

**Figure 1:**
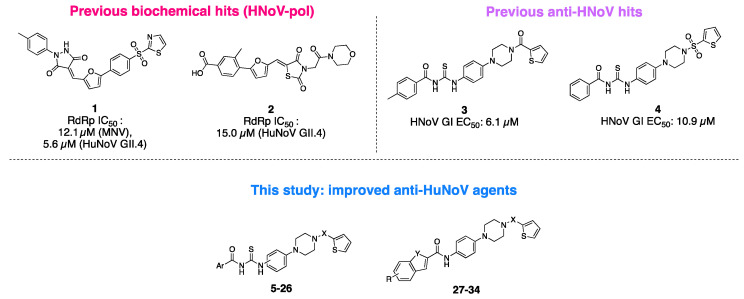
Previously identified biochemical (**1–2**) and antiviral (**3–4**) hits, and summary of the chemical modifications explored in this study.

Among the different potential targets for the development of anti-norovirus therapies, the viral polymerase represents a particularly promising one, due to its essential role for the viral replication [12], its prominent role as target in antiviral drug discovery [13], and its potential to provide an opportunity to optimize a broad-spectrum treatment [14]. As part of previous efforts to identify inhibitors of HuNoV replication, a virtual screening study of commercial libraries on the viral polymerase led to the identification of two biochemical hits, 1 and 2 in Figure 1 [15]. From these molecules, further computer-aided studies led to the design and synthesis of antiviral hits 3 and 4 (Figure 1), which inhibited the replication of human norovirus in cell-based assays with EC_50_ values in the low micromolar range [16]. Here, we present structure-activity relationship studies on the scaffolds of 3 and 4, which resulted in the identification of new low micromolar inhibitors of HuNoV replication in cellular systems. The new chemical modifications are summarized in Figure 1. In addition, we have a generated an initial 3D quantitative structure-activity relationship (3D-QSAR) model that, on top of revealing interesting trends for the antiviral potential of the scaffolds, could be used in the future for the design of improved antiviral agents to treat norovirus infections in humans.

## 2. Materials and Methods

### 2.1. Synthetic Chemistry

All solvents and reagents were used as obtained from commercial sources unless otherwise indicated. All solvents used for chromatography were HPLC grade from Fisher Scientific (Loughborough, UK). All reactions were performed under a nitrogen atmosphere. ^1^H and ^13^C-NMR spectra were recorded with a Bruker Avance III HD spectrometer operating at 500 MHz for ^1^H and 125 MHz for ^13^C, with Me_4_Si as internal standard (Bruker, Coventry, UK). Deuterated chloroform was used as the solvent for NMR experiments, unless otherwise stated. ^1^H chemical shifts values (δ) are referenced to the residual non-deuterated components of the NMR solvents (δ = 7.26 ppm for CHCl_3_, etc.). The ^13^C chemical shifts (δ) are referenced to CDCl_3_ (central peak, δ = 77.0 ppm). TLC was performed on silica gel 60 F254 plastic sheets. Flash column chromatography was performed using an Interchim automated system (Interchim, Montluçon, France). UPLC-MS analysis was conducted on a Waters UPLC system with both Diode Array detection and Electrospray (+′ve and −′ve ion) MS detection (Waters, Wilmslow, UK). The stationary phase was a Waters Acquity UPLC BEH C18 1.7 µm 2.1 × 50 mm column. The mobile phase was LC-MS grade H_2_O containing 0.1% formic acid (A) and LC-MS grade MeCN containing 0.1% formic acid (B). Column temperature: 40 ºC. Sample diluent: MeCN. Sample concentration 1 μg/mL. Injection volume 2 μL. Three alternative methods were used: Linear gradient standard method (A): 90% A (0.1 min), 90–0% A (2.5 min), 0% A (0.3 min), 90% A (0.1 min); flow rate 0.5 mL/min. Linear gradient standard method (B): 90% A (0.1 min), 90–0% A (2.1 min), 0% A (0.8 min), 90% A (0.1 min); flow rate 0.5 mL/min. Linear gradient standard method (C): 90% A (0.1 min), 90–0% A (1.5 min), 0% A (1.4 min), 90% A (0.1 min); flow rate 0.5 mL/min. All compounds tested in biological assays were >95% pure. Purity of intermediates was >90%, unless otherwise stated. All intermediates were prepared according to literature procedures, which are described in detail along with compound characterization in the Supporting Information. Details for the preparation and full characterization of the new target final compounds are given below.

#### 2.1.1. General Procedure for the Preparation of Target Products 5–26

The appropriate aryl chloride 50–58 (1 mmol) and ammonium thiocyanate (1 mmol) were dissolved in acetone (4 mL/mmol) at 0 ºC with vigorous stirring. Stirring was continued for 1 h at r.t., then the formed precipitate (NH_4_Cl) was filtered off. To the freshly filtered solution, the appropriate aromatic amine 44–49 (1 mmol) was added. The mixture was then stirred under reflux for 1 h. Upon completion of the reaction, the resulting precipitate was collected by filtration. The crude product was purified by trituration, re-crystallisation or automated flash column chromatography to afford the title compound.

- 4-(*tert*-Butyl)-*N*-((4-(4-(thiophen-2-ylsulfonyl)piperazin-1-yl)phenyl)carbamothioyl)benzamide (5)

Purified by automated flash column chromatography eluting with *n*-hexane:ethyl acetate 100:0 *v*/*v* increasing to 70:30 *v*/*v* in 5 CV. Obtained in 64% yield as a yellow solid. ^1^H-NMR (DMSO-d_6_), δ: 12.53 (s, 1H), 11.34 (s, 1H), 8.09 (dd, J_1_ = 4.9 Hz, J_2_= 1.3 Hz, 1H), 7.93 (d, J = 8.6 Hz, 2H), 7.70 (dd, J_1_ = 3.7 Hz, J_2_ = 1.3 Hz, 1H), 7.56 (d, J = 8.6 Hz, 2H), 7.52 (d, J = 9.1 Hz, 2H), 7.32 (dd, J_1_ = 4.9 Hz, J_2_ = 3.7 Hz, 1H), 6.96 (d, J = 9.1 Hz, 2H), 3.29–3.28 (m, 4H), 3.08–3.06 (m, 4H), 1.32 (s, 9H). ^13^C-NMR (DMSO-d_6_), δ: 179.1, 168.4, 156.7, 148.9, 134.8, 134.6, 133.8, 130.4, 129.7, 129.0, 128.9, 125.8, 125.6, 116.3, 48.1, 46.2, 35.3, 31.3. UPLC-MS (Method C): R_t_ 2.23 min, MS [ESI, *m*/*z*]: 543.1 [M + H].

- 4-(*tert*-Butyl)-*N*-((4-(4-(thiophene-2-carbonyl)piperazin-1-yl)phenyl)carbamothioyl)benzamide (6)

Purified by automated flash column chromatography eluting with *n*-hexane:ethyl acetate 100:0 *v*/*v* increasing to 50:50 *v*/*v* in 5 CV. Obtained in 68% yield as a yellow solid. ^1^H-NMR (DMSO-d_6_), δ: 12.55 (s, 1H), 11.35 (s, 1H), 7.94 (d, J = 8.6 Hz, 2H), 7.79 (dd, J_1_ = 5.1 Hz, J_2_ = 1.1 Hz, 1H), 7.56 (m, 4H), 7.48 (dd, J_1_ = 3.6 Hz, J_2_ = 1.1 Hz, 1H), 7.16 (dd, J_1_ = 5.1 Hz, J_2_ = 3.6 Hz, 1H), 7.00 (d, J = 9.1 Hz, 2H), 3.81–3.79 (m, 4H), 3.27–3.25 (m, 4H), 1.32 (s, 9H). ^13^C-NMR (DMSO-d_6_), δ: 176.6, 168.5, 162.8, 156.7, 149.3, 137.5, 130.1, 129.8, 129.6, 129.0, 127.6, 125.8, 125.6, 115.9, 55.3, 48.7, 35.3, 31.3. UPLC-MS (Method C): R_t_ 2.18 min, MS [ESI, *m*/*z*]: 507.2 [M + H].

- 4-Methoxy-*N*-((4-(4-(thiophen-2-ylsulfonyl)piperazin-1-yl)phenyl)carbamothioyl)benzamide (7)

Purified by automated flash column chromatography eluting with *n*-hexane:ethyl acetate 100:0 *v*/*v* increasing to 50:50 *v*/*v* in 15 CV. Obtained in 76% yield as a yellow solid. ^1^H-NMR (DMSO-d_6_), δ: 8.98 (s, 1H), 7.89–7.83 (m, 2H), 7.65 (dd, J_1_ = 5.0 Hz, J_2_ = 1.3 Hz, 1H), 7.60–7.55 (m, 3H), 7.17 (dd, J_1_ = 3.1 Hz, J_2_ = 3.7 Hz, 1H), 7.02–6.99 (m, 2H), 6.94 (d, j = 8.8 Hz, 2H), 3.89 (s, 3H), 3.33–3.31 (m, 4H), 3.26–3.25 (m, 4H). ^13^C-NMR (DMSO-d_6_), δ: 178.4, 176.6, 166.3, 164.0, 135.6, 132.7, 132.5, 129.6, 127.8, 125.3, 123.5, 116.9, 114.5, 55.6, 49.0, 45.9. UPLC-MS (Method C): R_t_ 2.01 min, MS [ESI, *m*/*z*]: 517.0 [M + H].

- 4-Methoxy-*N*-((4-(4-(thiophene-2-carbonyl)piperazin-1-yl)phenyl)carbamothioyl)benzamide (8)

Purified by automated flash column chromatography eluting with *n*-hexane:ethyl acetate 100:0 *v*/*v* increasing to 40:60 *v*/*v* in 15 CV. Obtained in 40% yield as a yellow solid. ^1^H-NMR (DMSO-d_6_), δ: 12.59 (s, 1H), 11.28 (s, 1H), 8.01 (d, J = 8.8 Hz, 2H), 7.79 (dd, J_1_ = 4.9 Hz, J_2_ = 0.9 Hz, 1H), 7.54 (d, J = 8.8 Hz, 2H), 7.48 (dd, J_1_ = 3.6 Hz, J_2_ = 0.9 Hz, 1H), 7.16 (dd, J_1_ = 4.9 Hz, J_2_ = 3.6 Hz, 1H), 7.07 (d, J = 8.9 Hz, 2H), 7.00 (d, J = 8.9 Hz, 2H), 3.86 (s, 3H), 3.81–3.79 (m, 4H), 3.26–3.24 (m, 4H). ^13^C-NMR (DMSO-d_6_), δ: 179.1, 167.9, 163.6, 162.8, 149.2, 137.5, 131.4, 130.1, 129.6, 127.6, 125.5, 124.4, 115.9, 114.2, 56.0, 48.8. UPLC-MS (Method C): R_t_ 1.93 min, MS [ESI, *m*/*z*]: 481.1 [M + H].

- *N*-((4-(4-(Thiophen-2-ylsulfonyl)piperazin-1-yl)phenyl)carbamothioyl)-4-(trifluoromethyl)benzamide (9)

Purified by automated flash column chromatography eluting with *n*-hexane:ethyl acetate 100:0 *v*/*v* increasing to 50:50 *v*/*v* in 10 CV. Obtained in 70% yield as a yellow solid. ^1^H-NMR (DMSO-d_6_), δ: 12.36 (s, 1H), 11.80 (s, 1H), 8.18 (d, J = 8.3 Hz, 2H), 8.14 (dd, J_1_ = 4.9 Hz, J_2_ = 1.3 Hz, 1H), 7.96 (d, J = 8.3 Hz, 2H), 7.75 (dd, J_1_ = 3.7 Hz, J_2_ = 1.3 Hz, 1H), 7.57 (d, J= 8.9 Hz, 2H), 7.37 (dd, J_1_ = 4.9 Hz, J_2_ = 3.7 Hz, 1H), 7.02 (d, j = 8.9 Hz, 2H), 3.35–3.33 (m, 4H), 3.14–3.12 (m, 4H). ^13^C-NMR (DMSO-d_6_), δ: 178.8, 176.6, 167.6, 148.9, 134.8, 134.6, 133.8, 133.2 (m), 130.3, 130.0, 128.9, 127.4 (m), 125.8 (m), 125.6, 116.3, 48.1, 46.2. UPLC-MS (Method C): R_t_ 2.09 min, MS [ESI, *m*/*z*]: 555.0 [M + H].

- *N*-((4-(4-(thiophene-2-carbonyl)piperazin-1-yl)phenyl)carbamothioyl)-4-(trifluoromethyl)benzamide (10)

Purified by automated flash column chromatography eluting with *n*-hexane:ethyl acetate 100:0 *v*/*v* increasing to 40:60 *v*/*v* in 15 CV. Obtained in 47% yield as a yellow solid. ^1^H-NMR (DMSO-d_6_), δ: 12.57 (s, 1H), 11.28 (s, 1H), 7.79–7.76 (m, 2H), 7.58 (dd, J_1_ = 6.3 Hz, J_2_ = 1.3 Hz, 1H), 7.51–7.48 (m, 3H), 7.11–7.09 (m, 1H), 6.94–6.91 (m, 2H), 6.84 (d, J = 8.9 Hz, 2H), 3.24–3.22 (m, 4H), 3.18–3.16 (m, 4H). ^13^C-NMR (DMSO-d_6_), δ: 178.4, 166.3, 164.0, 148.9, 135.6, 132.7 (m), 132.4, 130.7, 129.6, 127.8 (m), 125.3 (m), 123.5, 116.8, 114.5, 48.9, 45.9. UPLC-MS (Method C): R_t_ 2.03 min, MS [ESI, *m*/*z*]: 519.1 [M + H].

- 4-Fluoro-*N*-((4-(4-(thiophen-2-ylsulfonyl)piperazin-1-yl)phenyl)carbamothioyl)benzamide (11)

Re-crystallised from DCM/n-hexane. Obtained in 43% yield as a yellow solid. ^1^H-NMR (DMSO-d_6_), δ: 12.40 (s, 1H), 11.49 (s, 1H), 8.08 (dd, J_1_ = 4.9 Hz, J_2_ = 1.2 Hz, 1H), 7.69 (dd, J_1_ = 3.7 Hz, j_2_ = 1.2 Hz, 1H), 7.50 (d, J = 8.9 Hz, 2H), 7.38-7.34 (m, 4H), 7. 31 (dd, J_1_ = 4.9 Hz, J_2_ = 3.7 Hz, 1H), 6.96 (d, J = 8.9 Hz, 2H), 3.29–3.27 (m, 4H), 3.08–3.06 (m, 4H). ^13^C-NMR (DMSO-d_6_), δ: 179.0, 167.6, 166.3, 148.9, 134.8, 134.6, 133.8, 132.1, 130.4, 129.2, 128.9, 125.6, 116.3, 115.8, 48.1, 46.2. UPLC-MS (Method C): R_t_ 2.02 min, MS [ESI, *m*/*z*]: 505.1 [M + H].

- 4-Fluoro-*N*-((4-(4-(thiophene-2-carbonyl)piperazin-1-yl)phenyl)carbamothioyl)benzamide (12)

Purified by automated flash column chromatography eluting with *n*-hexane:ethyl acetate 100:0 *v*/*v* increasing to 0:100 *v*/*v* in 15 CV. Obtained in 93% yield as a yellow solid. ^1^H-NMR (DMSO-d_6_), δ: 12.40 (s, 1H), 9.01 (s, 1H), 7.93–7.90 (m, 2H), 7.61 (d, j= 8.9 Hz, 2H), 7.48 (dd, J_1_ = 5.1 Hz, J_2_ = 1.1Hz, 1H), 7.34 (dd, J_1_ = 3.6 Hz, J_2_ = 1.1 Hz, 1H), 7.24–7.20 (m, 2H), 7.07 (dd, J_1_ = 5.1 Hz, J_2_ = 3.6 Hz, 1H), 7.03 (d, J = 7.6 Hz, 2H), 3.99–3.94 (m, 4H), 3.30–3.28 (m, 4H). ^13^C-NMR (DMSO-d_6_), δ: 178.1, 176.6, 167.0, 165.7, 165.0, 163.7, 136.7, 130.2 (m), 129.1, 128.9, 127.8, 126.8, 125.3, 116.6 (m), 49.7, 46.1. UPLC-MS (Method C): R_t_ 1.94 min, MS [ESI, *m*/*z*]: 469.1 [M + H].

- Methyl.4-(((4-(4-(thiophen-2-ylsulfonyl)piperazin-1-yl)phenyl)carbamothioyl)carbamoyl)benzoate (13)

Purified by flash column chromatography eluting with *n*-hexane:ethyl acetate 100:0 *v*/*v* increasing to 40:60 *v*/*v* in 10 CV. Obtained in 42% yield as an off-white solid. ^1^H-NMR (CDCl_3_), δ: 12.39 (s, 1H), 9.06 (s, 1H), 8.20 (d, J = 8.57 Hz, 2H), 7.95 (d, J = 8.7 Hz, 2H), 7.66 (dd, J_1_ = 5.0 Hz, J_2_ = 1.3 Hz, 1H), 7.63 (d, J = 8.8 Hz, 2H), 7.59 (dd, J_1_ = 3.7 Hz, J_2_ = 1.3 Hz, 1H), 7.18 (dd, J_1_ = 5.0 Hz, J_2_ = 3.7 Hz, 1H), 7.06 (d, J = 8.8 Hz, 2H), 3.96 (s, 3H), 3.36–3.28 (m, 8H). ^13^C-NMR (CDCl_3_), δ: 177.9, 176.6, 166.0, 165.7, 163.4, 135.6, 135.3, 134.7, 132.8, 132.7, 130.3, 127.9, 127.5, 125.3, 117.7, 52.7, 49.9, 45.4. UPLC-MS (Method C): R_t_ 1.98 min, MS [ESI, *m*/*z*]: 545.0 [M + H].

- Methyl 4-(((4-(4-(thiophene-2-carbonyl)piperazin-1-yl)phenyl)carbamothioyl)carbamoyl)benzoate (14)

Purified by trituration from acetone/*n*-hexane. Obtained in 69% yield as an off-white solid. ^1^H-NMR (CDCl_3_), δ: 12.36 (s, 1H), 9.09 (s, 1H), 8.19 (d, J = 8.5 Hz, 2H), 7.95 (d, J = 8.5 Hz, 2H), 7.61 (d, J = 8.3 Hz, 2H), 7.48 (dd, J_1_ = 5.0 Hz, J_2_ = 1.1 Hz, 1H), 7.34 (dd, J_1_ = 3.6 Hz, J_2_ = 1.1 Hz, 1H), 7.07 (dd, J_1_ = 5.0 Hz, J_2_ = 3.6 Hz, 1H), 7.01 (d, J = 8.3 Hz, 2H), 3.97 (s, 3H), 3.30–3.28 (m, 8H). ^13^C-NMR (CDCl_3_), δ: 177.9, 176.6, 166.0, 165.3, 163.7, 144.7, 136.7, 135.4, 134.7, 130.3, 129.1, 128.9, 127.5, 126.8, 125.3, 116.7, 52.7, 49.6, 46.9. UPLC-MS (Method C): R_t_ 1.91 min, MS [ESI, *m*/*z*]: 509.1 [M + H].

- *N*-((4-(4-Thiophen-2-ylsulfonyl)piperazin-1-yl)phenyl)carbamothioyl)furan-2-carboxamide (15)

Purified by automated flash column chromatography eluting with *n*-hexane:dichloromethane 100:0 *v*/*v* increasing to 0:100 *v*/*v* in 10 CV. Obtained in 41% yield as a yellow solid. ^1^H-NMR (CDCl_3_), δ: 12.16 (s, 1H), 9.17 (s, 1H), 7.65 (dd, J_1_ = 5.0 Hz, J_2_ = 1.3 Hz, 1H), 7.62 (dd, J_1_ = 1.7 Hz, J_2_ = 0.8 Hz, 1H), 7.59–7.56 (m, 3H), 7.37 (dd, J_1_ = 3.6 Hz, J_2_ = 0.8 Hz, 1H), 7.17 (dd, J_1_ = 5.0 Hz, J_2_ = 3.7 Hz, 1H), 6.95 (d, J = 8.7 Hz, 2H), 6.63 (dd, J_1_ = 3.6 Hz, J_2_ = 1.7 Hz, 1H), 3.32–3.28 (m, 8H). ^13^C-NMR (CDCl_3_), δ: 177.6, 176.7, 163.4, 156.5, 144.7, 135.4, 146.2, 132.5, 132.2, 127.5, 125.1, 118.7, 116.6, 113.2, 48.7, 45.7. UPLC-MS (Method C): R_t_ 1.92 min, MS [ESI, *m*/*z*]: 477.1 [M + H].

- *N*-((4-(4-(Thiophene-2-carbonyl)piperazin-1-yl)phenyl)carbamothioyl)furan-2-carboxamide (16)

Purified by automated flash column chromatography eluting with *n*-hexane:ethyl acetate 100:0 *v*/*v* increasing to 0:100 *v*/*v* in 10 CV. Obtained in 51% yield as a yellow solid. ^1^H-NMR (CDCl_3_), δ: 12.18 (s, 1H), 9.18 (s, 1H), 7.62 (dd, J_1_ = 1.7 Hz, J_2_ = 0.8 Hz, 1H), 7.60 (d, J = 8.9 Hz, 2H), 7.48 (dd, J_1_ = 5.0 Hz, J_2_ = 1.1 Hz, 1H), 7.38 (dd, J_1_ = 3.6 Hz, J_2_ = 0.8 Hz, 1H), 7.34 (dd, J_1_ = 3.6 Hz, J_2_ = 1.1 Hz, 1H), 7.07 (dd, J_1_ = 5.0 Hz, J_2_= 3.6 Hz, 1H), 7.01 (d, J = 8.9 Hz, 2H), 6.63 (dd, J_1_ = 3.6 Hz, J_2_ = 1.7 Hz, 1H), 3.97–3.95 (m, 4H), 3.29–3.27 (m, 4H). ^13^C-NMR (CDCl_3_), δ: 177.6, 176.7, 163.4, 156.5, 144.7, 136.4, 135.7, 146.2, 128.9, 128.7, 126.6, 125.1, 118.6, 116.5, 113.2, 49.4, 46.5. UPLC-MS (Method C): R_t_ 1.83 min, MS [ESI, *m*/*z*]: 441.1 [M + H].

- *N*-((4-(4-(Thiophen-2-ylsulfonyl)piperazin-1-yl)phenyl)carbamothioyl)thiophene-2-carboxamide (17)

Purified by automated flash column chromatography eluting with dichloromethane:methanol 100:0 *v*/*v* increasing to 95:5 *v*/*v* in 10 CV. Obtained in 41% yield as a yellow solid. ^1^H-NMR (CDCl_3_), δ: 12.26 (s, 1H), 8.88 (s, 1H), 7.73–7.71 (m, 2H), 7.65 (dd, J_1_ = 5.1 Hz, J_2_ = 1.3 Hz, 1H), 7.60–7.58 (m, 3H), 7.20–7.17 (m, 2H), 7.00 (d, J = 8.7 Hz, 2H), 3.35–3.30 (m, 8H). ^13^C-NMR (CDCl_3_), δ: 177.9, 176.7, 161.1, 160.5, 136.2, 135.7, 134.4, 132.9, 132.6, 130.4, 128.6, 127.9, 125.4, 117.2, 49.4, 45.8. UPLC-MS (Method C): R_t_ 1.98 min, MS [ESI, *m*/*z*]: 493.1 [M + H].

- *N*-((4-(4-(Thiophene-2-carbonyl)piperazin-1-yl)phenyl)carbamothioyl)thiophene-2-carboxamide (18)

Purified by automated flash column chromatography eluting with dichloromethane:methanol 100:0 *v*/*v* increasing to 97:3 *v*/*v* in 10 CV. Obtained in 40% yield as a yellow solid. ^1^H-NMR (CDCl_3_), δ: 12.26 (s, 1H), 8.94 (s, 1H), 7.74–7.72 (m, 2H), 7.59 (d, J = 8.9 Hz, 2H), 7.48 (dd, J_1_ = 5.0 Hz, J_2_ = 1.1 Hz, 1H), 7.34 (dd, J_1_ = 3.6 Hz, J_2_ = 1.1 Hz, 1H), 7.19 (dd, J_1_ = 4.9 Hz, J_2_ = 3.8 Hz, 1H), 7.07 (dd, J_1_ = 5.0 Hz, J_2_ = 3.6 Hz, 1H), 7.00 (d, J = 8.9 Hz, 2H), 3.96–3.94 (m, 4H), 3.29–3.27 (m, 4H). ^13^C-NMR (CDCl_3_), δ: 177.9, 176.6, 163.7, 161.1, 144.7, 136.7, 136.0, 134.3, 130.7, 129.1, 128.9, 128.5, 126.8, 125.3, 116.7, 49.7, 47.1. UPLC-MS (Method C): R_t_ 1.90 min, MS [ESI, *m*/*z*]: 457.1 [M + H].

- 4-Methyl-*N*-((4-(4-(thiophen-2-ylsulfonyl)piperazin-1-yl)phenyl)carbamothioyl)benzamide (19)

Purified by automated flash column chromatography eluting with *n*-hexane:dichloromethane 100:0 *v*/*v* increasing to 40:60 *v*/*v* in 10 CV. Obtained in 66% yield as a yellow solid. ^1^H-NMR (DMSO-d_6_), δ: 12.51 (s, 1H), 11.36 (s, 1H), 8.09 (dd, J_1_ = 4.9 Hz, J_2_ = 1.1 Hz, 1H), 7.90 (dd, J_1_ = 3.6 Hz, J_2_ = 1.1 Hz, 1H), 7.70 (d, J = 8.9 Hz, 2H), 7.53–7.50 (m, 2H), 7.35–7.31 (m, 3H), 6.96 (d, J = 8.9 Hz, 2H), 3.29–3.27 (m, 4H), 3.08–3.06 (m, 4H), 2.39 (s, 3H). ^13^C-NMR (DMSO-d_6_), δ: 176.0, 167.6, 144.2, 143.5, 141.5, 132.0, 131.9, 129.8, 129.6, 129.5, 129.2, 128.9, 127.9, 125.6, 48.2, 48.0, 21.6. UPLC-MS (Method C): R_t_ 2.07 min, MS [ESI, *m*/*z*]: 501.1 [M + H].

- 4-Methyl-*N*-((3-(4-(thiophen-2-ylsulfonyl)piperazin-1-yl)phenyl)carbamothioyl)benzamide (20)

Purified by automated flash column chromatography eluting with dichloromethane:methanol 100:0 *v*/*v* increasing to 98:2 *v*/*v* in 10 CV. Obtained in 44% yield as an off-white solid. ^1^H-NMR (CDCl_3_), δ: 12.69 (s, 1H), 9.02 (s, 1H), 7.78 (d, J = 8.3 Hz, 2H), 7.66 (dd, J_1_ = 5.0 Hz, J_2_ = 1.3 Hz, 1H), 7.58 (dd, J_1_ = 3.7 Hz, J_2_= 1.3 Hz, 1H), 7.32 (m, 3H), 7.17 (dd, J_1_ = 5.0 Hz, J_2_ = 3.7 Hz, 1H), 7.15 (d, J = 6.6 Hz, 1H), 6.93 (d, J = 8.3 Hz, 2H), 3.38–3.25 (m, 8H), 2.46 (s, 3H). ^13^C-NMR (CDCl_3_), δ: 178.0, 176.6, 166.8, 144.9, 138.6, 135.6, 132.7, 132.5, 129.9, 129.6, 128.6, 127.8, 127.5, 116.9, 115.0, 112.4, 49.0, 45.8, 21.7. UPLC-MS (Method C): R_t_ 2.09 min, MS [ESI, *m*/*z*]: 501.1 [M + H].

- 4-Methyl-*N*-((3-(4-(thiophene-2-carbonyl)piperazin-1-yl)phenyl)carbamothioyl)benzamide (21)

Purified by automated flash column chromatography eluting with n-hexane:ethyl acetate 90:10 *v*/*v* increasing to 20:80 *v*/*v* in 15 CV. Obtained in 40% yield as an off-white solid. ^1^H-NMR (CDCl_3_), δ: 12.71 (s, 1H), 9.04 (s, 1H), 7.79 (d, J = 8.1 Hz, 2H), 7.70 (s, 1H), 7.48 (dd, J_1_ = 4.9 Hz, J_2_ = 0.8 Hz, 1H), 7.35–7.30 (m, 4H), 7.17 (d, J = 6.8 Hz, 1H), 7.07 (dd, J_1_ = 4.9 Hz, J_2_ = 3.6 Hz, 1H), 6.98 (m, 1H), 3.99 (m, 4H), 3.33–3.31 (m, 4H), 2.46 (s, 3H). ^13^C-NMR (CDCl_3_), δ: 178.0, 176.6, 176.1, 166.8, 163.7, 144.9, 138.6, 136.7, 129.9, 129.6, 129.1, 128.9, 128.6, 127.5, 126.8, 124.8, 112.1, 49.6, 46.5, 21.7. UPLC-MS (Method C): R_t_ 2.01 min, MS [ESI, *m*/*z*]: 465.1 [M + H].

- *N*-((3-(4-(Thiophen-2-ylsulfonyl)piperazin-1-yl)phenyl)carbamothioyl)benzamide (22)

Purified by automated flash column chromatography eluting with *n*-hexane:ethyl acetate 80:20 *v*/*v* increasing to 60:40 *v*/*v* in 10 CV. Obtained in 42% yield as a white solid. ^1^H-NMR (CDCl_3_), δ: 12.58 (s, 1H), 8.98 (s, 1H), 7.83–7.81 (m, 2H), 7.61–7.59 (m, 3H), 7.52 (dd, J_1_ = 3.7 Hz, J_2_ = 1.3 Hz, 1H), 7.50–7.46 (m, 2H), 7.26–7.23 (m, 1H), 7.12–7.07 (m, 2H), 6.89–6.86 (m, 1H), 3.32–3.28 (m, 4H), 3.27–3.25 (m, 4H). ^13^C-NMR (CDCl_3_), δ: 178.1, 176.7, 138.8, 135.8, 135.5, 134.0, 132.9, 132.6, 131.7, 129.9, 129.4, 127.9, 127.6, 115.5, 112.7, 49.4, 45.9. UPLC-MS (Method C): R_t_ 2.06 min, MS [ESI, *m*/*z*]: 487.1 [M + H].

- *N*-((3-(4-(Thiophene-2-carbonyl)piperazin-1-yl)phenyl)carbamothioyl)benzamide (23)

Purified by automated flash column chromatography eluting with *n*-hexane:ethyl acetate 80:20 *v*/*v* increasing to 60:40 *v*/*v* in 7 CV. Obtained in 53% yield as a white solid. ^1^H-NMR (CDCl_3_), δ: 12.68 (s, 1H), 9.10 (s, 1H), 7.90–7.86 (m, 3H), 7.68–7.61 (m, 2H), 7.57–7.54 (m, 2H), 7.49–7.48 (m, 1H), 7.37– 7.33 (m, 2H), 7.21–7.19 (m, 1H), 7.10–7.06 (m, 1H), 4.04–3.99 (m, 4H), 3.37–3.33 (m, 4H). ^13^C-NMR (CDCl_3_), δ: 179.1, 176.9, 170.4, 139.2 136.0, 135.8, 135.6, 134.9, 133.2, 132.6, 129.5, 129.2, 127.3, 126.9, 114.8, 112.3, 49.2, 45.8. UPLC-MS (Method C): R_t_ 1.95 min, MS [ESI, *m*/*z*]: 451.2 [M + H].

- 4-Methyl-*N*-((2-(4-(thiophen-2-ylsulfonyl)piperazin-1-yl)phenyl)carbamothioyl)benzamide (24)

Purified by automated flash column chromatography eluting with dichloromethane:methanol 100:0 *v*/*v* increasing to 98:2 *v*/*v* in 5 CV. Obtained in 53% yield as a white solid. ^1^H-NMR (DMSO-d_6_), δ: 13.20 (s, 1H), 8.84 (m, 2H), 8.18 (dd, J_1_ = 5.1 Hz, J_2_ = 1.3 Hz, 1H), 7.70–7.68 (m, 3H), 7.41–7.40 (m, 2H), 7.38–7.36 (m, 1H), 7.33–7.31 (m, 1H), 7.23–7.20 (m, 2H), 3.16–3.14 (m, 4H), 2.99–2.97 (m, 4H), 2.5 (s, 3H). ^13^C-NMR (DMSO-d_6_), δ: 177.7, 167.6, 144.2, 143.9, 135.0, 133.9, 129.1, 134.4, 133.7, 129.5, 129.3, 128.8, 126.5, 124.7, 122.4, 121.6, 51.3, 46.6, 21.6. UPLC-MS (Method C): R_t_ 2.05 min, MS [ESI, *m*/*z*]: 501.1 [M + H].

- *N*-((2-(4-(Thiophen-2-ylsulfonyl)piperazin-1-yl)phenyl)carbamothioyl)benzamide (25)

Purified by automated flash column chromatography eluting with *n*-hexane:dichloromethane 90:10 *v*/*v* increasing to 0:100 *v*/*v* in 7 CV. Obtained in 64% yield as a white solid. ^1^H-NMR (DMSO-d_6_), δ: 13.07 (s, 1H), 8.88–8.85 (m, 2H), 7.64–7.61 (m, 3H), 7.56 (dd, J_1_ = 5.6 Hz, J_2_ = 1.3 Hz, 1H), 7.53 (dd, 1H, J_1_ = 3.7 Hz, J_2_ = 1.3 Hz, 1H), 7.48–7.47 (m, 2H), 7.16–7.14 (m, 3H), 7.12–7.10 (m, 1H), 3.37–3.35 (m, 4H), 2.98–2.96 (m, 4H). ^13^C-NMR (DMSO-d_6_), δ: 176.5, 165.9, 143.8, 136.8, 134.2, 132.1, 134.2, 133.1, 132.4, 129.5, 128.1, 128.0, 127.1, 125.7, 122.7, 121.4, 52.1, 46.8. UPLC-MS (Method C): R_t_ 2.11 min, MS [ESI, *m*/*z*]: 487.1 [M + H].

- *N*-((2-(4-(thiophene-2-carbonyl)piperazin-1-yl)phenyl)carbamothioyl)benzamide (26)

Purified by automated flash column chromatography eluting with *n*-hexane:ethyl acetate 80:20 *v*/*v* increasing to 60:40 *v*/*v* in 10 CV. Obtained in 41% yield as a white solid. ^1^H-NMR (DMSO-d_6_), δ: 13.37 (s, 1H), 8.79 (dd, J_1_ = 8.2 Hz, J_2_ = 1.5 Hz, 1H), 8.09–8.06 (m, 1H) 7,81 (dd, J_1_ = 5.0 Hz, J_2_ = 1.5 Hz, 1H), 7.85– 7.83 (m, 1H), 7.79 (dd, J_1_ = 8.2 Hz, J_2_ = 5.0 Hz, 1H), 7.70–7.68 (m, 1H) 7.60–7.56 (m, 2H), 7.51 (dd, J_1_ = 3.7 Hz, J_2_ = 1.3 Hz, 1H), 7.35 (dd, J_1_ = 6.3 Hz, J_2_ = 1.3 Hz, 1H), 7.29–7.26 (m, 1H), 7.24–7.21 (m, 1H), 7.18–7.16 (m, 1H), 3.91 (m, 4H), 2.96 (m, 4H). ^13^C-NMR (DMSO-d_6_), δ: 178.0, 176.6, 168.6, 162.8, 144.5, 137.5, 132.7, 130.0, 129.6, 129.2, 128.8, 127.5, 126.7, 124.3, 123.2, 121.3, 52.3, 46.9. UPLC-MS (Method C): R_t_ 1.99 min, MS [ESI, *m*/*z*]: 451.2 [M + H].

#### 2.1.2. General Procedure for the Preparation of Target Products 27–34

The appropriate aromatic amine 44 or 45 (0.5 mmol), the appropriate carboxylic acid 68-73 (0.6 mmol), TBTU (0.6 mmol) and DMF (3 mL/mmol) were added to a 10 mL round-bottom flask equipped with a septum and nitrogen balloon. Et_3_N (0.6 mmol) was added dropwise to the mixture while stirring and stirring was continued at room temperature under an N_2_ atmosphere until completion of the reaction (5–48 h), which was monitored by TLC. The reaction mixture was then diluted with EtOAc (20 mL) and H_2_O (10 mL), and the organic layer was washed with sat. NaHCO_3_ (10 mL), H_2_O (10 mL) and brine (10 mL), filtered over Na_2_SO_4_ and dried under *vacuum*. The crude product was purified by trituration or re-crystallisation to afford the title compound.

- *N*-(4-(4-(Thiophen-2-ylsulfonyl)piperazin-1-yl)phenyl)benzofuran-2-carboxamide (27)

Purified by trituration from ethyl acetate. Obtained in 45% yield as an off-white solid. ^1^H-NMR (DMSO-d_6_), δ: 10.36 (s, 1H), 8.09 (dd, J_1_ = 5.1 Hz, J_2_ = 1.3 Hz, 1H), 7.81 (ddd, J_1_ = 7.9 Hz, J_2_ = 1.3 Hz, J_3_ = 0.8 Hz, 1H), 7.71-7.69 (m, 3H), 7.66 (d, J = 9.2 Hz, 2H), 7.49 (ddd, J_1_= 8.1 Hz, J_2_= 7.2 Hz, J_3_ = 1.3 Hz, 1H), 7.36 (ddd, J_1_ = 7.9 Hz, J_2_ = 7.2 Hz, J_3_ = 0.8 Hz, 1H), 7.32 (dd, J_1_ = 5.1 Hz, J_2_ = 3.7 Hz, 1H), 6.94 (d, J = 9.2 Hz, 2H), 3.25–3.22 (m, 4H), 3.09–3.06 (m, 4H). ^13^C-NMR (DMSO-d_6_), δ: 156.6, 154.8, 149.4, 147.3, 134.8, 134.6, 133.8, 131.3, 128.8, 127.6, 127.4, 124.2, 123.8, 122.0, 116.8, 112.3, 110.6, 48.5, 46.2. UPLC-MS (Method C): R_t_ 2.27 min, MS [ESI, *m*/*z*]: 468.1 [M + H].

- *N*-(4-(4-(Thiophene-2-carbonyl)piperazin-1-yl)phenyl)benzofuran-2-carboxamide (28)

Purified by trituration from ethyl acetate. Obtained in 52% yield as an off-white solid. ^1^H-NMR (DMSO-d_6_), δ: 10.30 (s, 1H), 7.82 (ddd, J_1_ = 7.9 Hz, J_2_ = 1.3 Hz, J_3_ = 0.7 Hz, 1H), 7.79 (dd, J_1_ = 5.0, J_2_ = 1.1 Hz, 1H), 7.72-7.70 (m, 2H), 7.69 (d, J= 9.1, 2H), 7.50 (ddd, J_1_ = 9.8 Hz, J_2_ = 7.1 Hz, J_3_ = 1.3 Hz, 1H), 7.47 (dd, J_1_ = 3.6 Hz, J_2_ = 1.1 Hz, 1H), 7.37 (ddd, J_1_ = 7.9 Hz, J_2_ = 7.1 Hz, J_3_ = 0.7 Hz, 1H), 7.16 (dd, J_1_ = 5.0 Hz, J_2_ = 3.6 Hz, 1H), 6.99 (d, J = 9.1, 2H), 3.81–3.79 (m, 4H), 3.21–3.19 (m, 4H). ^13^C-NMR (DMSO-d_6_), δ: 162.8, 156.6, 154.8, 149.5, 147.8, 137.5, 131.5, 130.0, 129.6, 127.7, 127.6, 127.4, 124.3, 123.2, 122.1, 116.5, 112.3, 110.6, 49.3, 49.2. UPLC-MS (Method C): R_t_ 2.14 min, MS [ESI, *m*/*z*]: 432.1 [M + H].

- 5-Methoxy-*N*-(4-(4-(thiophene-2-carbonyl)piperazin-1-yl)phenyl)benzofuran-2-carboxamide (29)

Purified by trituration from ethyl acetate. Obtained in 47% yield as an off-white solid. ^1^H-NMR (DMSO-d_6_), δ: 10.35 (s, 1H), 7.79 (dd, J_1_ = 5.0 Hz, J_2_ = 1.1 Hz, 1H), 7.68 (d, J = 9.1 Hz, 2H), 7.65 (bs, 1H), 7.61 (ddd, J_1_ = 9.0 Hz, J_2_ = 0.6 Hz, J_3_ = 0.6 Hz, 1H), 7.47 (dd, J_1_ = 3.6 Hz, J_2_ = 1.1 Hz, 1H), 7.30 (d, J = 2.4 Hz, 1H), 7.16 (dd, J_1_ = 5.0 Hz, J_2_ = 3.6 Hz, 1H), 7.08 (dd, J_1_ = 9.0 Hz, J_2_ = 2.6 Hz, 1H), 6.98 (d, J = 9.1 Hz, 2H), 3.82 (s, 3H), 3.81–3.79 (m, 4H), 3.21–3.18 (m, 4H). ^13^C-NMR (DMSO-d_6_), δ: 162.8, 159.6, 156.5, 150.1, 149.7, 147.7, 137.5, 130.0, 129.6, 128.3, 127.6, 122.1, 116.7, 112.9, 110.7, 104.6, 56.1, 49.3, 49.2. UPLC-MS (Method C): R_t_ 2.12 min, MS [ESI, *m*/*z*]: 462.1 [M + H].

- *N*-(4-(4-(Thiophen-2-ylsulfonyl)piperazin-1-yl)phenyl)-1*H*-indole-2-carboxamide (30)

Purified by trituration from H_2_O. Obtained in 59% yield as a white solid. ^1^H-NMR (DMSO-d_6_), δ: 11.69 (s, 1H), 10.05 (s, 1H), 8.09 (dd, J_1_ = 5.0 Hz, J_2_ = 1.3 Hz, 1H), 7.70 (dd, J_1_ = 3.7 Hz, J_2_ = 1.3 Hz, 1H), 7.67–7.63 (m, 3H), 7.47 – 7.43 (m, 1H), 7.35 (d, J = 1.3 Hz, 1H), 7.32 (dd, J_1_ = 5.0 Hz, J_2_ = 3.7 Hz, 1H), 7.20 (ddd, J_1_ = 8.8 Hz, J_2_ = 7.8 Hz, J_3_ = 0.9 Hz, 1H), 7.06 (ddd, J_1_ = 8.8 Hz, J_2_ = 7.8 Hz, J_3_ = 0.9 Hz, 1H), 6.94 (d, J = 9.1 Hz, 2H), 3.25–3.22 (m, 4H), 3.09–3.07 (m, 4H). ^13^C-NMR (DMSO-d_6_), δ: 157.4, 154.3, 149.1, 147.7, 137.8, 133.8, 132.6, 131.4, 129.7, 127.8, 127.4, 124.7, 123.8, 121.7, 116.5, 113.8, 112.3, 48.4, 46.5. UPLC-MS (Method C): R_t_ 2.21 min, MS [ESI, *m*/*z*]: 467.1 [M + H].

- *N*-(4-(4-(Thiophene-2-carbonyl)piperazin-1-yl)phenyl)-1*H*-indole-2-carboxamide (31)

Purified by trituration from ethyl acetate. Obtained in 69% yield as an off-white solid. ^1^H-NMR (DMSO-d_6_), δ: 11.69 (d, J = 1.7 Hz, 1H), 10.07 (s, 1H), 7.79 (dd, J_1_ = 5.0 Hz, J_2_ = 1.1 Hz, 1H), 7.68–7.65 (m, 3H), 7.48–7.45 (m, 2H), 7.34 (dd, J_1_= 2.1 Hz, J_2_= 0.7 Hz, 1H), 7.21 (ddd, J_1_ = 8.3 Hz, J_2_ = 6.9 Hz, J_3_= 1.1 Hz, 1H), 7.16 (dd, J_1_ = 5.5 Hz, J_2_ = 3.16 Hz, 1H), 7.06 (ddd, J_1_ = 7.9 Hz, J_2_ = 6.9 Hz, J_3_ = 0.9 Hz, 1H), 7.00 (d, J = 9.1 Hz, 2H), 3.81 – 3.7 (m, 4H), 3.21 – 3.17 (m, 4H). ^13^C-NMR (DMSO-d_6_), δ: 170.8, 162.8, 159.7,147.5, 137.5, 137.1, 132.2, 131.7, 130.0, 129.6, 127.6, 124.0, 122.0, 121.7, 120.2, 116.6, 112.7, 103.8, 49.5, 49.4. UPLC-MS (Method C): R_t_ 2.09 min, MS [ESI, *m*/*z*]: 431.2 [M + H].

- 5-Methyl-*N*-(4-(4-(thiophen-2-ylsulfonyl)piperazin-1-yl)phenyl)-1*H*-indole-2-carboxamide (32)

Purified by trituration from H_2_O. Obtained in 55% yield as a light red solid. ^1^H-NMR (DMSO-d_6_), δ: 11.54 (s, 1H), 10.01 (d, J = 6.0 Hz, 1H), 8.09 (dd, J_1_ = 5.0 Hz, J_2_ = 1.3 Hz, 1H) 7.70 (dd, J_1_ = 3.7 Hz, J_2_ = 1.3 Hz, 1H), 7.64 (d, J = 9.1 Hz, 2H), 7.44 – 7.42 (m, 1H), 7.36–7.32 (m, 2H), 7.26 (d, J = 1.3 Hz, 1H), 7.03 (dd, J_1_ = 8.2 Hz, J_2_ = 1.3 Hz, 1H), 6.94 (d, J = 9.1 Hz, 2H), 3.24–3.21 (m, 4H), 3.09–3.06 (m, 4H), 2.39 (s, 3H). ^13^C-NMR (DMSO-d_6_), δ: 158.3, 157.7, 150.5, 148.7, 147.5, 139.5, 131.6, 130.1, 129.2, 124.3, 123.4, 120.1, 116.4, 110.2, 109.6, 49.3, 48.7, 23.5. UPLC-MS (Method C): R_t_ 2.30 min, MS [ESI, *m*/*z*]: 481.1 [M + H].

- 5-Methoxy-*N*-(4-(4-(thiophen-2-ylsulfonyl)piperazin-1-yl)phenyl)-1*H*-indole-2-carboxamide (33)

Purified by trituration from H_2_O. Obtained in 45% yield as an off-white solid. ^1^H-NMR (DMSO-d_6_), δ: 11.53 (s, 1H), 10.05 (s, 1H), 8.10 (ddd, J_1_ = 5.0 Hz, J_2_= 1.3 Hz, J_3_ = 0.4 Hz, 1H), 7.70 (ddd, J_1_ = 3.7 Hz, J_2_ = 1.3 Hz, J_3_ = 0.4 Hz, 1H), 7.63 (dd, J_1_ = 5.2 Hz, J_2_ = 2.0 Hz 2H), 7.36 – 7.32 (m, 2H), 7.26 (d, J= 2.2 Hz, 1H), 7.12 (d, J= 2.2 Hz, 1H), 6.95 (dd, J_1_ = 7.0 Hz, J_2_ = 2.2 Hz, 2H), 6.86 (dd, J_1_ = 8.8 Hz, J_2_ = 2.2 Hz, 1H), 3.78 (s, 3H), 3.25–3.22 (m, 4H), 3.10–3.07 (m, 4H). ^13^C-NMR (DMSO-d_6_), δ: 159.4, 156.5, 152.2, 149.1, 146.6, 140.7, 132.2, 131.8, 130.5, 125.0, 123.2, 119.4, 116.8, 111.6, 110.5, 57.4, 49.5, 48.9. UPLC-MS (Method C): R_t_ 2.18 min, MS [ESI, *m*/*z*]: 497.1 [M + H].

- 6-Methoxy-*N*-(4-(4-(thiophen-2-ylsulfonyl)piperazin-1-yl)phenyl)-1*H*-indole-2-carboxamide (34)

Purified by trituration from ethyl acetate. Obtained in 47% yield as an off-white solid. ^1^H-NMR (DMSO-d_6_), δ: 11.50 (s, 1H), 9.94 (s, 1H), 8.09 (dd, J_1_ = 4.9 Hz, J_2_ = 1.3 Hz, 1H), 7.70 (dd, J_1_ = 3.7 Hz, J_2_ = 1.3 Hz, 1H), 7.62 (d, J= 9.1 Hz, 2H), 7.53 (d, J = 8.7 Hz, 1H), 7.32 (dd, J_1_ = 4.9 Hz, J_2_ = 3.7 Hz, 1H), 7.29 (d, J = 1.3 Hz, 1H), 6.95 (d, J = 9.1 Hz, 2H), 6.90 (d, J = 2.1 Hz, 1H), 6.72 (dd, J_1_ = 8.7, Hz, J_2_ = 2.1 Hz, 1H), 3.76 (s, 3H), 3.23–3.21 (m, 4H), 3.08–3.06 (m, 4H). ^13^C-NMR (DMSO-d_6_), δ: 159.8, 157.7, 146.9, 138.1, 134.9, 134.6, 133.8, 132.3, 131.0, 128.9, 122.9, 121.8, 121.6, 117.0, 111.6, 104.1, 94.5, 55.5, 48.7, 46.3. UPLC-MS (Method C): R_t_ 2.18 min, MS [ESI, *m*/*z*]: 497.1 [M + H].

### 2.2. Antiviral Assays

Cells and virus: MNV (virus strain MNV-1.CW1) was propagated in RAW 264.7 cells grown in DMEM (Life Technologies, Gent, Belgium) supplemented with 10% or 2% FBS, 2 mM L-glutamine, 20 mM HEPES, 0.075 g/L sodium bicarbonate, 1 mM sodium pyruvate, 100 U penicillin/mL and 100 lg/mL streptomycin at 37 ºC in a humidified atmosphere of 5% CO_2_. The human norovirus GI.1 replicon harboring gastric tumor-1 cell line (HGT-NV) (kindly provided by Dr. Ian Goodfellow, University of Cambridge) was maintained in DMEM supplemented with 10% FBS, 2mM L-glutamine, 0.075 g/L sodium bicarbonate and 1.0 mg/mL of Geneticin (G418; Life Technologies), at 37 ºC in a humidified atmosphere of 5% CO_2_ [16].

Antiviral assay with MNV: The antiviral activity of the compounds was determined using an MTS [3-(4,5-dimethylthiazol-2-yl)-5-(3-carboxymethoxyphenyl)-2-(4-sulfophenyl)-2H-tetrazolium]-based cytopathic effect (CPE) reduction assay. RAW 264.7 cells (1 × 10^4^ cells/well) were seeded in a 96-well plate and infected with MNV (MOI of 0.001) in the presence (or absence) of a dilution series of compounds. Cells were incubated for 3 days, i.e., until complete CPE was observed in infected untreated cells. Then, a MTS-phenazinemethosulfate (MTS/PMS) stock solution [(2 mg/mL MTS (Promega, Leiden, The Netherlands) and 46 g/mL PMS (Sigma–Aldrich, Bornem, Belgium) in PBS at pH 6–6.5)] was diluted 1/20 in MEM (Life Technologies, Gent, Belgium) and 75 μL were added to each well. After 2 h, the optical density (OD) was read at 498 nm. The %CPE reduction was calculated as [(OD_treated_)_MNV_ − OD_VC_]/[OD_CC_ − OD_VC_] x 100, where OD_CC_ represents the OD of the uninfected untreated cells, whereas OD_VC_ and (OD_treated_)_MNV_ represent the OD of infected untreated cells and virus-infected cells treated with a compound concentration, respectively. The 50% effective concentration (EC_50_) was defined as the compound concentration that protected 50% of the cells from virus-induced CPE [16]. 

Cytotoxicity: The cytotoxicity of the compounds was evaluated by the MTS method, by exposing uninfected cells to the same concentrations of compounds for 3 days. The %cell viability was calculated as (OD_treated_/OD_CC_) × 100, where OD_CC_ is the OD of uninfected untreated cells and OD_treated_ are uninfected cells treated with compound. The CC_50_ was defined as the compound concentration that reduces the number of viable cells by 50% [16].

Antiviral assay with the HGT-NV replicon: The inhibitory effect of compounds on human norovirus replication was assessed by quantification of the levels of HuNoV GI replicon RNA and of the reference (housekeeping) gene β-actin mRNA [by reverse transcription quantitative polymerase chain reaction (RT-qPCR)]. To this end, HGT cells (2000 cells/well) were seeded in 96-well plates, in complete DMEM without the selection marker G418. Following an incubation period of 24 h, a serial dilution of rupintrivir was added to the cultures. After 72 h of incubation, cell monolayers were washed with phosphate-buffered saline (PBS) and collected for quantification of RNA load by RT-qPCR. Intracellular RNA was extracted from cells using the cell-to-cDNA lysis buffer (Ambion, Life Technologies, Gent, Belgium). For detection of HuNoV GI replicon RNA, forward (5′-CCG GCT ACC TGC CCA TTC-3′), reverse (5′-CCA GAT CAT CCT GAT CGA CAA G-3′) primers and probe (5′-FAM-ACA TCG CAT CGA GCG AGC ACG TAC-TAMRA-3′) for the neomycin gene were used. For detection of β-actin mRNA, forward (5′-GGC ATC CAC GAA ACT ACC TT-3′), reverse (5′-AGC ACT GTG TTG GCG TAC AG-3′) primers and probe (5′-HEX-ATC ATG AAG TGT GAC GTG GAC ATC CG-BHQ1–3′) were used. One-step RT-qPCR was performed in a 20 μL reaction mixture containing 10 μL 2X iTaq Universal SYBR Green one step reaction mix (Bio-rad, Hercules, CA, USA), 5.17 μL RNAse free water, 0.5 μL of iScript reverse transcriptase (Bio-rad, California, USA), 4 μL of the template RNA and either 300 nM of HuNoV GI replicon primers and probe, 300 nM of β-actin primers and 200 nM of probe. Cycling conditions were: reverse transcription at 50 °C for 10 min, initial denaturation at 95 °C for 3 min, followed by 40 cycles of denaturation at 95 °C for 15 s, annealing and extension at 60 °C for 30 s (Roche Lightcycler 96, Roche Diagnostics, Brusselles, Belgium). To determine the relative expression levels of HuNoV GI replicon RNA, β-actin was used as a normalizer and ratios were calculated using the Pfaffl method. The Expression Ratio (HuNoV GI Replicon/β-actin) was calculated as: Expression Ratio = (E_HuNoV_)^ΔCT, HuNoV (CC – TC)^/(E_β-actin_)^ΔCT, β-actin (CC – TC)^, where E_HuNoV_ and E_β-actin_ represent the amplification efficiency (E = 10^−1/slope^) for the HuNoV GI replicon and β-actin RT-qPCR reactions, respectively. ΔCT, HuNoV (CC – TC) is the Ct of untreated control cells (CC) minus the Ct of cells treated with a compound concentration (TC) obtained with HuNoV GI replicon primers and probe. ΔCT, β-actin (CC – TC) is the Ct of untreated control cells (CC) minus the Ct of cells treated with a compound concentration (TC) obtained with β-actin primers and probe. Efficiency values (E_HuNoV_ and E_β-actin_) were determined for each RT-qPCR reaction. The 50% effective concentration (EC_50_) was defined as the compound concentration that resulted in a 50% reduction of the relative HuNoV replicon RNA levels [16].

### 2.3. Molecular Modelling

All molecular modelling studies were performed on a Viglen Genie Intel^®^ Core^TM^ i7–3770 vPro CPU@ 3.40 GHz x 8 running Ubuntu 16.04. Molecular Operating Environment (MOE) 2020.10 [17] and Forge v10 software (Cresset Inc., Cambridgeshire, UK) [18] were used as molecular modelling software. The Flexible Alignment was performed using MOE 2020.10. The MOE flexible alignment tool generates different possible conformations for each molecule present in the input database that could overlap the conformation of the assigned template, which is kept rigid (compounds 3 and 4). The quality of the alignment is evaluated by a score which is a sum of the internal strain of the obtained conformation (the smaller, the better) and the overlap of molecular features (aromatic regions, donors/acceptors). MOE, for each alignment performed, evaluates the average internal energy of the ligands U, the similarity score F (the lower the value is, the better the two structures overlap) and the value S (sum of U and F values obtained for each alignment). A good alignment should present a dU value (the average strain energy of the molecules in the alignment in kcal/mol) lower than 1 kcal/mol meaning that the obtained conformations are not energetically disadvantaged. All the newly designed compounds (**27–34**) are predicted to have an optimal structural overlapping with compound 3 or 4, having dU of 0.0 (no energy penalty) and low S values.

The conformation of 3 and 4 required for the Flexible Alignment was obtained using MOE conformational search tool, using the default parameters, selecting Stochastic as search method, and setting the RMS Gradient to 0.001 and the RMSD Limit to 0.15. 130 conformations per each compound were generated and among them the best in terms of dE (the strain of the conformation relative to the lowest energy conformation with the same stereochemistry configuration; value of 0 indicate the best result) and E (value of the potential energy of the conformation) were chosen as template for the Flexible Alignment.

The 2D structure of compounds 27–34 were drawn using Marvin version 21.17.0 [19] and then converted in a .mdb data base in MOE.

FieldTemplater module of Forge v10 software (Cresset Inc., Cambridgeshire, UK) was used to determine a hypothesis for the 3D pharmacophore model by comparing the electrostatic and hydrophobic properties of the most active compounds (8, 30, 32, 34, and 4 for MNV, and 27, 28, and 30 for HuNoV). Each compound was then aligned to generate a pharmacophore query, one for each virus. The alignments were performed to maximise similarity in terms of molecular field and molecular shape similarity. Field point-based descriptors were used for building the 3D-QSAR model after the alignment of the molecules present in the data set. The data set were randomly partitioned to put 20% in the test set and leaving the remaining molecules in the training set. The experimental activity (EC_50_) of the data set compounds were converted to its positive-logarithmic scale using the formula: pEC_50_ = −log (EC_50_) and defined as the dependent variable. The 3D-QSAR was constructed based on the aligned molecules, and it was assessed by the LOO technique to optimise the activity prediction model. A list of all the compounds used for this calculation is reported in the Supporting Information.

## 3. Results and Discussion

### 3.1. Design and Synthesis of Novel Antiviral Analogues

**Scheme 1:**
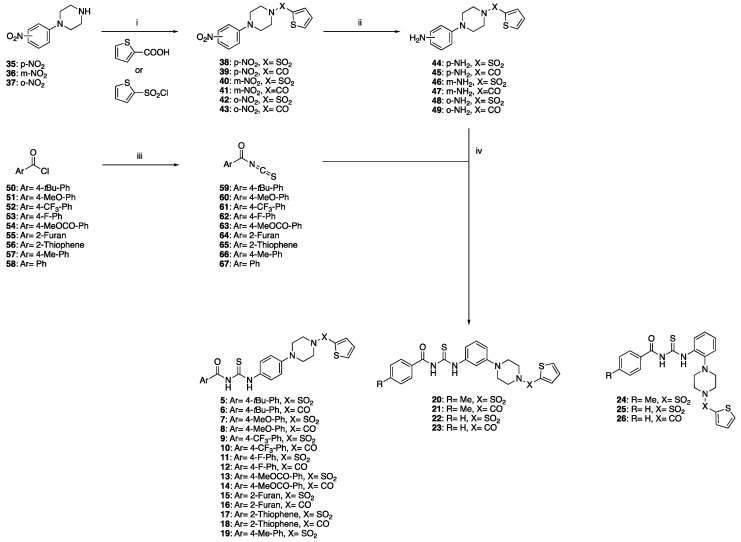
Synthesis of target compounds 5–26. *Reagents and conditions:* (i) TBTU, DiPEA, DMF, r.t., 5 h, OR Et_3_N, DCM, 0 °C to r.t., o.n., 80–99%; (ii) H_2_, 10% Pd/C, EtOH, r.t., 24 h, 75–99%; (iii) CNSH*NH_3_, Me_2_CO, reflux, 1 h, quant.; (iv) Me_2_CO, reflux, 2 h, 40–93%.

With the aim to further explore structure-activity relationships and improve the antiviral properties of our previous hits 3 and 4, a first series of novel analogues 5–26 was designed, to insert different substituents at the *para* position of the terminal phenyl ring (Ar group) of the hit scaffolds (5–14), as the presence of a methyl substituent in this position had previously been found associated with activity retention [15]. In parallel, two heteroaromatic rings were also explored as replacements of the phenyl group in this position, a 2-furan and 2-thiophene ring (15–18). In our previous studies, we had already confirmed the presence of a 2-thiophene ring next to the amide or sulfonamide terminal group as essential for antiviral activity, therefore this portion of the scaffold was not altered in this study. However, we also wanted to investigate the importance of the overall three-dimensional shape of the molecules, by changing the substitution pattern at the central phenyl ring from *para*-substituted (3–4) to *meta* (20–23) and *ortho* (24–26). Finally, novel analogue 19 was also prepared, to allow for a more systematic activity comparison between the two scaffolds of 3 (thiophene-amide) and 4 (thiophene-sulfonamide). The novel target compounds 5–26 were prepared according to a four-step synthetic route we previously described [15], summarized in Scheme 1.

Following this route, commercial nitrophenyl-piperazines **35–37** were treated with either thiophene-2-carboxylic acid or thiophene-2-sulfonyl chloride to give intermediate nitro-compounds 38–43. Amide-intermediates 39, 41, 43 were obtained through a TBTU-assisted coupling reaction in the presence of DiPEA, stirring the reaction in DMF at r.t.. Sulfonamide intermediates 38, 40, 42 were instead obtained in DCM at 0 °C to r.t., in the presence of Et_3_N as a base. Nitro-intermediates 38–43 were subsequently converted into the corresponding substituted aromatic amines 44–49 following a catalytic hydrogenation in EtOH, in the presence of wet activated palladium on carbon. Isothiocyanates 59–67 were obtained by treating the corresponding aryl-carbonyl chlorides 50–58 with ammonium isothiocyanate, refluxing the reaction in acetone. These isothiocyanates were not isolated, and treated *in situ* with aromatic amines 44–49, to give the desired final products 5–26, in variable yields. As previously observed [15], in different cases the yield of the final reaction step was lowered by the formation of unwanted amide byproducts.

**Figure 2:**
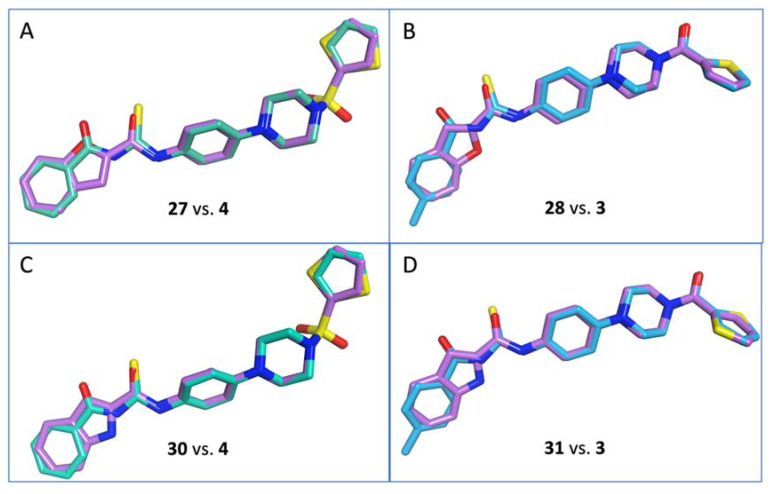
Flexible Alignment results for compounds 27, 28, 30 and 31 (carbon atoms in lilac) against either 3 (carbon atoms in light blue) or 4 (carbon atoms in green), showing the high degree of structural overlapping between the newly designed compounds and the original hit scaffolds.

A second series of novel analogues, 27–34, was envisaged to remove the thioamide portion of the amide-thioamide linker between the terminal phenyl group (Ar) and the central phenyl ring in the structures of 3 and 4, while maintaining the overall length of the scaffold. Removal of the thioamide portion was mainly planned to overcome the synthetic problems encountered with the amide-thioamide linker generation [15]. Structural analogues where this central amide-thioamide linker has been replaced by a simple amide, previously obtained as byproducts of the final reaction step in Scheme 1 [15], were associated with loss of antiviral activity [15], possibly due to the molecular scaffold being significantly shortened. In an attempt to remove the thioamide component of the amide-thioamide linker, while preserving the overall length of the molecule, the insertion of bicyclic heteroaromatic rings to replace the terminal phenyl group (Ar) was envisaged. Considering commercially available bicyclic, heteroaromatic carboxylic acids, a selection of six possible substitution patterns was made, to include an unsubstituted benzoxazole (27–28) or indole moiety (30–31), and rings with a 5-methoxy, 5-methyl or 6-methoxy substituents (29, 32, 33, 34), to mimic the presence of the original *para*-methyl group in the terminal phenyl ring of 3. The potential of the newly designed analogues to maintain the same molecular length and overall occupational space of 3 and 4 was confirmed by running a flexible alignment analysis with the Flexible Alignment tool in MOE 2020.10 [17]. For this analysis, a conformational search was first performed on 3 and 4, using MOE conformational search tool [17], to identify their lowest energy conformation. This conformation was then kept rigid for each hit scaffold, and the newly designed compounds 27–34 were checked against it by the software exploring their conformational space, to identify potential three-dimensional and functional similarities (overlapping). Figure 2 shows the results obtained from this analysis for selected compounds, which highlight how the newly designed molecules are predicted to have an optimal structural overlapping with either hit 3 (for 28 and 31) or 4 (for 27 and 30), conserving the overall molecular length.

**Scheme 2:**
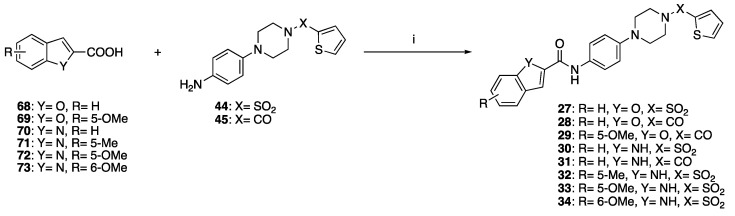
Synthesis of target compounds **27–34**. *Reagents and conditions:* (i) TBTU, Et_3_N, DMF, r.t., 5–48 h, 45–69%.

Once their structural superimposition with the original hits 3 and 4 was confirmed, the novel target molecules 27–34 were prepared by reacting substituted aniline intermediates 44–45 with commercially available acids 68–73, according to a TBTU-assisted amide coupling reaction, using Et_3_N as a base, as shown in Scheme 2.

### 3.2. Cell-Based Antiviral Studies against MNV and HuNoV

The thirty novel molecules prepared 5–34 were evaluated for their antiviral effect against the genogroup V mouse virus (MNV), using the mouse macrophage cell line RAW264.7. This assay evaluates the ability of the compounds to protect infected cells from the virus-induced cytopathic effect, thus enabling to identify inhibition of virus replication at every step of the virus life cycle and providing a robust cellular assay for the rapid evaluation of potential norovirus inhibitors. Compounds identified using MNV have shown effects against HuNoV in vitro and in vivo, as in the case of nucleoside RdRp inhibitor 2′-C-methylcytidine (2CMC) [20,21], which was included as positive control. The test compounds were evaluated at eight different concentrations (range 0.6–100 μM), and their ability to reduce the virus-induced cytopathic effect was assessed. All the novel compounds were also tested against HuNoV using a replicon system, a human gastric tumor-1 (HGT-1) cell line which stably expresses the genome of a HuNoV GI.1 virus [21]. In this system the gene encoding for the major capsid protein is replaced by a neomycin resistance gene, therefore no new virus particles can be produced, but the non-structural proteins are expressed, and the replication of the genomic RNA can be studied. Rupintrivir was included as positive control for this assay [22]. The results obtained, along with the compounds’ cytotoxicity in both systems, are summarized in Table 1.

Considering the results obtained for both the murine (MNV) and human (HuNoV) viruses, the trend observed is different for the two viruses, as previously found for 3 and 4 and their analogues. The different *para*-modifications explored in 5–19 for the terminal phenyl ring (Ar) are associated with loss of antiviral activity in the MNV, with the only exceptions being amide derivatives 8 and 14, bearing a methoxy and a carboxymethyl ester respectively, which retain antiviral properties similar to 3 and 4. In general, only few substituents seem to be tolerated in this position. Similarly, only sulfonamide derivative 13, bearing a carboxymethyl ester substituent, retains a comparable antiviral activity to the parent compounds in the HuNoV. More interesting results are instead obtained for the exploration of the role of the overall molecular shape for antiviral potential (20–26). While changing the substitution pattern at the central phenyl ring of the scaffold from *para* (3–4) to *meta* (20–23) is associated with loss of activity, the *ortho* substitution (24–26) appears instead to be associated with activity retention, especially for the inhibition of the replication of HuNoV, as can be observed from the data obtained for 25 and 26. These results are in contrast with our previous speculations that a linear scaffold is essential for antiviral activity, and, due to the overall structural rigidity of the compounds, they may suggest either a completely different binding mode to the same target, or binding to (and inhibition of) a different target. Additional investigations to further expand this structural series and to clarify the mechanism(s) of antiviral action of these compounds are currently ongoing.

Data obtained for the novel analogues 27–34 against MNV confirm an overall retention of antiviral potential against the mouse virus, with a better activity profile for the indole derivatives (30–34), and no associated toxicity. Most interestingly, analogues 27, 28, 30 and 31 display antiviral activities against HuNoV in the low micromolar range, with 31 reaching sub-micromolar EC_50_ values. Compounds 30 and 31 possess an interesting activity profile against both murine and human norovirus. Among the structural modifications explored, there appears to be a clear trend, as the presence of a methyl or methoxy substituent in the condensed phenyl ring of all scaffolds (29, 32–34) seems to be detrimental for activity, while both modifications explored at the level of the five-membered condensed ring (furan or pyrrole) are tolerated. However, the most active novel derivatives (27, 28, 31) are also associated with an increased cytotoxicity in the human cell line, as indicated by their lowered CC_50_ values compared to the parent molecules. While further synthetic efforts are currently ongoing to expand this promising series of compounds, our priority will be to identify new analogues with reduced cytotoxicity and improved selectivity indexes.

The antiviral data obtained have been further analyzed with computational methods and used to build an initial 3D-QSAR model, as described below.

### 3.3. 3D-QSAR Studies

As already mentioned, the starting point of this work was the identification of two biochemical hits, 1 and 2 (Figure 1), active against the viral polymerase, which did not possess a significant antiviral effect in cell-based assays [14]. Further studies led to the design and synthesis of antiviral hits 3 and 4 (Figure 1), which inhibited the replication of human norovirus in cell-based assays with EC_50_ values in the low micromolar range [15]. Consequently, for any new compound developed, the effect on cell-based assays was prioritized, with the aim to improve antiviral activity and develop potential antiviral agents, rather than biochemical hits. To facilitate the investigation of antiviral structure-activity relationships for the new compounds presented here, and to design more potent analogues in the future, an initial 3D quantitative structure-activity relationship (3D-QSAR) model was developed. This model was created using the software Forge v10 (Cresset Inc., UK) [18]. All the synthesised molecules were included in the 3D-QSAR model with their respective EC_50_ values (expressed as pEC_50_) and were randomly partitioned to put 20% in the test set, leaving the remaining molecules in the training set. The Cresset 3D-QSAR analyzed the diverse molecular fields of each molecule, such as positive and negative electrostatic fields, van der Waals shape, hydrophobic fields, and their spatial distribution [18]. The molecules in both training and test set are aligned to a reference template according to the calculated field points, to find a common field point distribution. In the absence of certainty on the compounds’ viral target and of a known inhibitor crystal structure in complex with the target, the most active compounds were used to derive a pharmacophore model, by comparing the different conformations of each molecule and identifying common electrostatic and hydrophobic motifs. The pharmacophore model was then used as a template to align all the remaining molecules in both the training and test set. During the 3D-QSAR modelling, the maximum number of conformations produced for each compound was set to 500, whereas the maximum distance for the sample point set to 0.5 Å. The predictive ability of the derived 3D-QSAR model was confirmed by leave-one-out (LOO) technique, analysing the r^2^, q^2^ value and the root-mean-square error (RMSE) of the predictive model. For this study, our previously developed analogues of 3 and 4 were also included [15]. A list of all molecules considered, along with their activity and cytotoxicity data, can be found in the Supporting Information (Tables S1 and S2).

**Figure 3:**
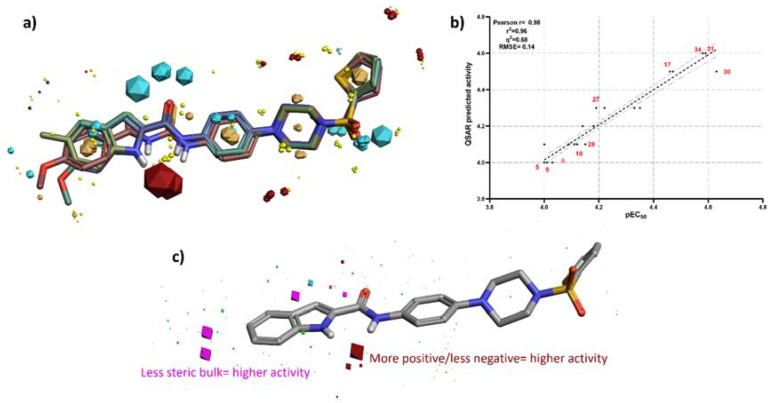
3D-QSAR results for murine norovirus. (**a**) Pharmacophore template with the respective molecular field points generated using compounds 8, 30, 32 and 34; (**b**) Plot of experimental (expressed as pEC_50_) vs. predicted activity of the 3D-QSAR model; (**c**) The visual representation of the coefficients and variance field points modulating the bioactivity in the derived 3D-QSAR model.

Although our main interest is developing new antivirals against human norovirus, and the generation of a reliable 3D-QSAR model can facilitate this process, we also investigated an initial 3D-QSAR model for the murine virus to identify, if any, potential interesting structure-activity patterns. Compounds 8, 30, 32 and 34 were used to generate a pharmacophore template for the murine virus (Figure 3a). The remaining 41 compounds were randomly partitioned in the test set (8 molecules) and the training set (33 molecules) (Supporting Information, Table S1). The generated 3D-QSAR model possesses good predictive and descriptive capabilities, as demonstrated by the good r^2^ (0.98) and q^2^ (0.68) values, and by the low RMSE (0.14) (Figure 3b). Figure 3c shows the 3D visualisation of the field 3D-QSAR model generated, applied to compound 30. Among all the different field points forming this 3D-QSAR model (coloured dots on Figure 3c), two major steric/electrostatic features seem to have a major role for the biological activity of this family of compounds against murine norovirus: an unfavourable steric spatial occupation, as indicated by the large size of magenta fields points, and a favourable positive electrostatic contribution, as highlighted by the large red field point. Overall, the 3D-QSAR results indicate that a bulky hindered group at the para position of the terminal phenyl ring Ar (compounds 5–26) is not well tolerated and causes a loss of activity, as seen when a trifluoromethyl or tert-butyl group were introduced in the structures of 3 and 4 (3 vs. 6 and 10; 4 vs. 5 and 9). Similarly, the model suggests that substituents larger than a methyl or methoxy group in position 5 or 6 of the bicyclic heteroaromatic ring (compounds 27–34) might have the same detrimental effect. Localization of a positive electrostatic area in correspondence of the amide-thioamide linker (compounds 5–26) or of the five-ring heteroatom-amide linker, seems important for the antiviral activity. This feature is in line with the better activity profile found for indole derivatives over benzofuran derivatives (a nitrogen atom is less electronegative than an oxygen, 27 vs. 30, 28 vs. 31) in the MNV assay. Combination/presence of these two features can positively affect antiviral activity.

**Figure 4:**
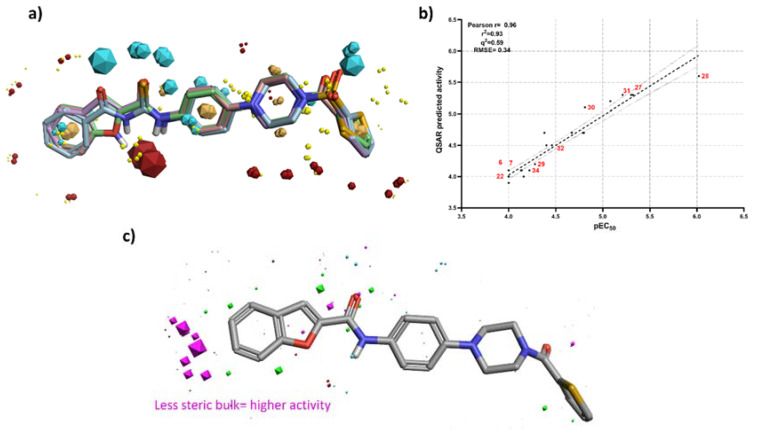
3D-QSAR results for human norovirus. (**a**) Pharmacophore template with the respective molecular field points generated using compounds 4, 27, 28 and 30; (**b**) Plot of experimental (expressed as pEC_50_) vs. predicted activity of the 3D-QSAR model; (**c**) The visual representation of the coefficients and variance field points modulating the bioactivity in the derived 3D-QSAR model.

Compounds 4, 27, 28 and 31 were used to prepare a pharmacophore template for activity against HuNoV (Figure 4a). Following the same approach adopted for the murine virus, a new 3D-QSAR model was generated (36 compounds were randomly partitioned in the test set, 7 molecules, and the training set 29 molecules) (Supporting Information, Table S2). The model possesses a good correlation between the predicted activity and the experimental EC_50_, but with a higher RMSE, potentially indicating that more data need to be collected to optimise it (Figure 4b). Interestingly, as in the murine model, an important feature that seems favourable for antiviral activity is the absence of bulky groups in both the terminal phenyl ring Ar (compounds 5–26) and the bicyclic heteroaromatic ring (compounds 27–34). Specific electrostatic contribution in correspondence of the amide-thioamide linker (compounds 5–26) or of the five-ring heteroatom-amide linker, seems not be required for antiviral activity. Although this new model requires further validation and refinement, it can now be employed for the prediction of antiviral activity against HuNoV of newly designed molecules, thus aiding the prioritization of future synthetic efforts for the most promising compounds, likely to be associated with a retained or improved antiviral potential.

## 4. Conclusions

Starting from the structures of two antiviral hit compounds previously identified as potential treatments for norovirus infections, a series of ligand-based modifications have been designed to explore SARs of these chemical scaffolds. Thirty novel analogues were synthesized according to a three- or four-step synthetic route, and subsequently evaluated in cell-based antiviral assays for their inhibition of the replication of both murine (MNV) and human (HuNoV) norovirus. Among the novel compounds evaluated, different interesting low-micromolar inhibitors of HuNoV replication were identified, possessing moderate toxicity. The biological data obtained have been then employed to build an initial 3D-QSAR model, which can now be used to direct the rational design of new potential inhibitors and, at the same time, can be further refined and optimized using new biological data generated. The new improved compounds are now being evaluated in other different human norovirus replication models, such as organoids and zebrafish larvae. Based on these findings, additional investigations on these new structures are currently ongoing to identify suitable preclinical antiviral candidates for the treatment of norovirus infections.

## Figures and Tables

**Table 1:** Antiviral (MNV CPE and HuNoV GI replicon) and cytotoxicity data for the novel analogues 5–34. Results obtained for hits **3–4** are also included.

Comp.	Structure	MNV EC_50_^A^ (µM)	MNV CC_50_^A^ (µM)	HuNoV EC_50_^A^ (µM)	HuNoV CC_50_^A^ (µM)
	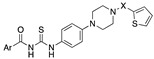				
**3**	**Ar = 4-Me-Ph, X = CO**	**46.6 ± 0.3**	**>100**	**15.8 ± 13.5**	**64.5 ± 31.5**
**4**	**Ar= Ph, X= SO_2_**	**44.4 ± 9.5**	**>100**	**4.8 ± 5.2**	**63.9 ± 22.5**
**5**	Ar = 4-*t*Bu-Ph, X = SO_2_	>100	78.80 ± 27.5	>100	>100
**6**	Ar = 4-*t*Bu-Ph, X = CO	>100	80.5 ± 33.8	>100	>100
**7**	Ar = 4-MeO-Ph, X = SO_2_	81.1 ± 30.1	>100	>100	>100
**8**	Ar = 4-MeO-Ph, X = CO	26.1 ± 11.3	>100	39.5 ± 52.4	8.44 ± 3.73
**9**	Ar = 4-CF_3_-Ph, X = SO_2_	97.5 ± 4.9	>100	73.5 ± 46.0	11.21 ± 6.31
**10**	Ar = 4-CF_3_-Ph, X = CO	75.3 ± 29.1	>100	>100	11.67 ± 9.06
**11**	Ar = 4-F-Ph, X = SO_2_	>100	59.9 ± 31.4	21.4 ± 9.6	15.26 ± 11.12
**12**	Ar = 4-F-Ph, X = CO	>100	>100	41.3 ± 50.9	29.53 ± 14.16
**13**	Ar = 4-MeOCO-Ph X = SO_2_	>100	>100	16.4 ± 13.3	>100
**14**	Ar = 4-MeOCO-Ph X = CO	46.6 ± 12.1	>100	>100	>100
**15**	Ar = 2-Furan, X = SO_2_	66.0 ± 35.6	>100	58.7 ± 36.9	63.50 ± 31.81
**16**	Ar = 2-Furan, X = CO	>100	>100	>100	67.71 ± 24.49
**17**	Ar = 2-Thiophene X = SO_2_	34.7 ± 18.1	>100	45.4 ± 47.3	17.82 ± 5.00
**18**	Ar = 2-Thiophene, X = CO	60.5 ± 29.2	>100	>100	>100
**19**	Ar = 4-Me-Ph, X = SO_2_	>100	49.8 ± 35.6	71.7 ± 49.1	37.42 ± 11.84
	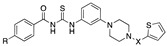				
**20**	R = Me, X = SO_2_	78.2 ± 27.3	>100	>100	>100
**21**	R = Me, X = CO	92.7 ± 9.2	>100	>100	81.3 ± 37.5
**22**	R= H, X = SO_2_	99.0 ± 1.7	>100	>100	>100
**23**	R = H, X = CO	>100	>100	>100	68.8 ± 37.5
	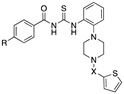				
**24**	R = Me, X = SO_2_	34.0 ± 30.1	>100	80.4 ± 23.5	>100
**25**	**R = H, X = SO_2_**	**77.5 ± 15.4**	**>100**	**17.4 ± 16.2**	**>100**
**26**	**R = H, X = CO**	**72.9 ± 46.9**	**>100**	**9.4 ± 4.2**	**87.5 ± 25.0**
	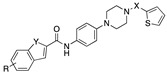				
**27**	R = H, Y = O, X = SO_2_	**64.3 ± 41.3**	**>100**	**5.0 ± 1.9**	**22.0 ± 7.4**
**28**	R = H, Y = O, X = CO	**70.8 ± 30.2**	**>100**	**0.9 ± 0.4**	**4.1 ± 1.7**
**29**	R = 5-OMe, Y = O, X = CO	>100	93.2 ± 11.8	52.5 ± 32.1	87.5 ± 25.0
**30**	R = H, Y = NH, X = SO_2_	23.7 ± 12.4	>100	15.6 ± 3.3	70.0 ± 21.7
**31**	R = H, Y = NH, X = CO	**25.9 ± 14.2**	**>100**	**6.2 ± 4.4**	**18.2 ± 9.3**
**32**	R = 5-Me, Y = NH, X = SO_2_	25.8 ± 14.6	>100	34.9 ± 6.1	>100
**33**	R = 5-OMe, Y = NH X = SO_2_	25.5 ± 14.3	>100	68.9 ± 37.0	>100
**34**	R = 6-OMe, Y = NH X = SO_2_	26.2 ± 14.7	>100	60.4 ± 30.2	>100
**2CMC**	Positive control	16.2 ± 5.9	36.5 ± 7.7	n.d.^B^	n.d.^B^
**Rupintrivir**	Positive control	n.d.^B^	n.d.^B^	1.5 ± 0.2	>100

The most interesting antiviral results obtained are highlighted in bold. ^A^ The mean values ± standard deviations are shown from at least three independent experiments. ^B^ Not determined.

## Data Availability

Not applicable.

## Supplementary Materials

The following are available online at https://www.mdpi.com/article/10.3390/microorganisms9091795/s1: synthetic procedures and characterization of intermediates 38–43, 44–49.

## References

[1] Robilotti E., Deresinski S., Pinsky B.A. (2015). Norovirus. Clin. Microbiol. Rev..

[2] Desselberger U. (2017). Viral gastroenteritis. Medicine.

[3] Kumthip K., Khamrin P., Maneekarn N. (2019). Epidemiology and genotypic distribution of noroviruses in patients with acute gastroenteritis in developing and developed countries. Ann. Res. Hosp..

[4] Bartsch S.M., Lopman B.A., Ozawa S., Hall A.J., Lee B.Y. (2016). Global Economic Burden of Norovirus Gastroenteritis. PLoS ONE.

[5] Randazzo W., D’Souza D.H., Sanchez G. (2018). Norovirus: The burden of the unknown. Adv. Food Nutr. Res..

[6] Chhabra P., De Graaf M., Parra G.I., Chan M.C.-W., Green K., Martella V., Wang Q., White P.A., Katayama K., Vennema H. (2019). Updated classification of norovirus genogroups and genotypes. J. Gen. Virol..

[7] Kim Y., Galasiti Kankanamalage A.C., Chang K.O., Groutas W.C. (2015). Recent Advances in the Discovery of Norovirus Therapeutics. J. Med. Chem..

[8] Esposito S., Principi N. (2020). Norovirus Vaccine: Priorities for Future Research and Development. Front. Immunol..

[9] Netzler N.E., Tuipulotu D.E., White P.A. (2019). Norovirus antivirals: Where are we now?. Med. Res. Rev..

[10] Chimerix (2018). Chimerix Announces Discovery and Demonstrated Preclinical Activity Supporting Ongoing Phase 1 Study of New Antiviral for Treatment and Prevention of Norovirus. http://ir.chimerix.com/news-releases/news-release-details/chimerix-announces-discovery-and-demonstrated-preclinical.

[11] Lanier R.S.D., Kolawole A., Hosmillo M., Nayak K., Bae A., Gurley S., Tippin T., Colton H., Dunn J., Mullin M. CMX521: A nucleoside with pan-genotypic activity against norovirus. Proceedings of the 31st International Conference on Antiviral Research.

[12] Adamson C.S., Chibale K., Goss R.J.M., Jaspars M., Newman D.J., Dorrington R.A. (2021). Antiviral drug discovery: Preparing for the next pandemic. Chem. Soc. Rev..

[13] Bassetto M., Van Dycke J., Neyts J., Brancale A., Rocha-Pereira J. (2019). Targeting the viral polymerase of diarrhea-causing viruses as a strategy to develop a single broad-spectrum antiviral therapy. Viruses.

[14] Ferla S., Netzler N.E., Ferla S., Veronese S., Tuipulotu D.E., Guccione S., Brancale A., White P.A., Bassetto M. (2018). In silico screening for human norovirus antivirals reveals a novel non-nucleoside inhibitor of the viral polymerase. Sci. Rep..

[15] Giancotti G., Rigo I., Pasqualetto G., Young M.T., Neyts J., Rocha-Pereira J., Brancale A., Ferla S., Bassetto M. (2019). A new antiviral scaffold for human norovirus identified with computer-aided approaches on the viral polymerase. Sci. Rep..

[16] Rocha-Pereira J., Jochmans D., Debing Y., Verbeken E., Nasvimento M.S.J., Neyts J. (2013). The viral polymerase Inhibitor 2′-C-Methylcytidine inhibits norwalk virus replication and protects against norovirus-induced diarrhea and mortality in a mouse model. J. Virol..

[17] Molecular Operating Environment (MOE 2020.10). Chemical Computing Group, Inc.: Montreal, QC, Canada. http://www.chemcomp.com.

[18] Forge, Version 10, Cresset®, Litlington, Cambridgeshire, UK. http://www.cresset-group.com/forge/.

[19] ChemAxon. https://www.chemaxon.com.

[20] Kolawole A.O., Rocha-Pereira J., Elftman M.D., Neyts J., Wobus C.E. (2016). Inhibition of human norovirus by a viral polymer-ase inhibitor in the B cell culture system and in the mouse model. Antivir. Res..

[21] Kitano M., Hosmillo M., Emmott E., Lu J., Goodfellow I. (2018). Selection and characterization of rupintrivir-resistant norwalk virus replicon cells in vitro. Antimicrob. Agents Chemother..

[22] Rocha-Pereira J., Nascimento M.S.J., Ma Q., Hilgenfeld R., Neyts J., Jochmans D. (2014). The enterovirus protease inhibitor rupintrivir exerts cross-genotypic anti-norovirus activity and clears cells from the norovirus replicon. Antimicrob. Agents Chemother..

