# Supplementary material for: Structure–Activity Relationship Studies on Novel Antiviral Agents for Norovirus Infections
Source: Microorganisms. 2021 Aug 24;9(9):1795. doi: 10.3390/microorganisms9091795 (PMC8468020; doi:10.3390/microorganisms9091795)
Supplement: Supplementary file 1 [file microorganisms-09-01795-s001.zip › microorganisms-1332239-supplementary.pdf]

Supplementary Information

Structure-activity relationship studies on novel antiviral agents for norovirus infections

Salvatore Ferla [1], Carmine Varricchio [2], William Knight [3], Pui Kei Ho [2], Fabiana Saporito [2], Beatrice Tropea [2], Giulio Fagan [2], Ben Matthew Flude [3], Federica Bevilacqua[2], Nanci Santos-Fereirra [4], Jana Van Dycke [4], Johan Neyts [4], Andrea Brancale [2,*], Joana Rocha-Pereira [4], and Marcella Bassetto [3,*]

[1] Swansea University Medical School, Swansea SA28PP, UK
[2] School of Pharmacy and Pharmaceutical Sciences, Cardiff University, Cardiff CF10 3NB, UK
[3] Department of Chemistry, Faculty of Science and Engineering, Swansea University, Swansea SA28PP, UK
[4] Rega Institute for Medical Research, University of Leuven, Belgium
* Correspondence: MB: marcella.bassetto@swansea.ac.uk; AB: brancalea@cardiff.ac.uk

Contents

Page S2 Preparation and characterisation of synthetic intermediates

Page S4 Table S.1 Murine 3D-QSAR model data

Page S6 Table S.2 Human 3D-QSAR model data

Page S7 References

S.1 General method for the preparation of sulfonamides 38, 40, 42

The appropriate nitrophenyl-piperazine **35-37** (2.41 mmol, 1 eq.) was dissolved in DCM (15 mL), EtN_3 (1.2 eq.) was added and the reaction was cooled to 0 °C. The appropriate thiophene-2-sulfonyl chloride (1.1 eq.) was dissolved in 5 mL DCM and the solution was added dropwise to the reaction mixture. The reaction was stirred at 0 °C for 1 hour, then allowed to warm to room temperature and stirred overnight. Upon completion of the reaction, saturated aqueous $NaHCO_3$ (20 mL) was added and the reaction was extracted with DCM (3x 25 mL). The organic phase was evaporated at reduced pressure and the crude residue was purified by flash column chromatography or trituration to afford the desired compounds.

S.1.1 1-(4-Nitrophenyl)-4-(thiophen-2-ylsulfonyl)piperazine (**38**)[S1]

Purified by trituration from EtOAc/n-hexane. Obtained in 99% yield as a yellow solid. ^{1}H-NMR (DMSO-d_6), δ: 8.09 (d, J= 9.5 Hz, 2H), 7.80 (dd, J_1= 5.0 Hz, J_2= 1.1 Hz, 1H), 7.50 (dd, J_1= 3.6 Hz, J_2= 1.1 Hz, 1H), 7.16 (dd, J_1= 5.0 Hz, J_2= 3.6 Hz, 1H), 7.01 (d, J= 9.5 Hz, 2H), 3.83 (m, 4H), 3.62-3.60 (m, 4H). ^{13}C-NMR (DMSO-d_6), δ: 154.6, 138.1, 135.0, 134.7, 133.8, 128.9, 126.1, 113.7, 46.2, 45.8.

S.1.2 1-(3-Nitrophenyl)-4-(thiophen-2-ylsulfonyl)piperazine (**40**)

Purified by automated flash column chromatography eluting with *n*-hexane:DCM 70:30 v/v increasing to 10:90 v/v in 10 CV. Obtained in 80% yield as a yellow solid. ^{1}H-NMR (DMSO-d_6), δ: 7.84 (dd, J_1= 6.5 Hz, J_2= 1.5 Hz, 1H), 7.77 (dd, J_1= 5.0 Hz, J_2= 1.1 Hz, 1H), 7.63-7.60 (m, 1H), 7.47 (dd, J_1= 3.5 Hz; J_2= 1.1 Hz, 1H), 7.38-7.37 (m, 1H), 7.20-7.17 (m, 1H), 7.15-7.13 (m, 1H), 3.78-3.76 (m, 4H), 3.09-3.07 (m, 4H).

S.1.3 1-(2-Nitrophenyl)-4-(thiophen-2-ylsulfonyl)piperazine (**42**)

Purified by automated flash column chromatography eluting with *n*-hexane:DCM 70:30 v/v increasing to 10:90 v/v in 10 CV. Obtained in 87% yield as a yellow solid. ^{1}H-NMR ($CDCl_3$), δ: 7.77 (dd, J_1= 5.1 Hz, J_2= 1.3 Hz, 1H), 7.66 (dd, J_1= 6.5 Hz, J_2= 1.5 Hz, 1H), 7.57 (dd, J_1= 3.7 Hz, J_2= 1.3 Hz, 1H), 7.53-7.50 (m, 1H), 7.18-7.16 (m, 2H), 7.16-712 (m, 1H), 3.24-3.20 (m, 4H), 3.17-3.13 (m, 4H).

S.2 General method for the preparation of amides 39, 41, 43

The appropriate nitrophenyl-piperazine **35-37** (2.41 mmol, 1 eq.), thiophene-2-carboxylic acid (2.65 mmol) and TBTU (2.89 mmol) were dissolved in dry DMF (7 mL) under N_2 atmosphere. Then, DiPEA (1.05 mL, 6.02 mmol) was added to the mixture and the reaction was stirred at room temperature for 5h. The solution was then partitioned between EtOAc (100 mL) and saturated aqueous NH_4Cl (70 mL). The organic phase was re-extracted once with NH_4Cl (70 mL), dried over Na_2SO_4 and concentrated under vacuum. The crude residue was purified by flash column chromatography or trituration to afford the desired compounds.

S.2.1 (4-(4-Nitrophenyl)piperazin-1-yl)(thiophen-2-yl)methanone (**39**)[S1]

Purified by trituration from EtOAc/*n*-hexane. Obtained in 94% yield as a yellow solid. ^{1}H-NMR (DMSO-d_6), δ: 8.09 (d, J= 9.5 Hz, 2H), 7.80 (dd, J_1= 6.0 Hz, J_2=1.1 Hz, 1H), 7.50 (dd, J_1= 3.7 Hz, J_2= 1,1 Hz, 1H), 7.17 (dd, J_1= 6.0 Hz, J_2= 3.7 Hz, 1H), 7.00 (d, J= 9.5 Hz, 2H), 3.83- 3.82 (m, 4H), 3.63-3.61 (m, 4H).

S.2.2 (4-(3-Nitrophenyl)piperazin-1-yl)(thiophen-2-yl)methanone (**41**)

Purified by automated flash column chromatography eluting with n-hexane:DCM 50:50 v/v increasing to 0:100 v/v in 10 CV. Obtained in 85% yield as a red solid. ^{1}H-NMR ($CDCl_3$), δ: 7.74 (dd, J_1= 8.1 Hz, J_2= 1.5 Hz, 1H), 7.47-7.42 (m, 1H), 7.40 (dd, J_1= 3.8 Hz, J_2= 1.5 Hz, 1H), 7,25 (dd, J_1= 2.5 Hz, J_2= 1.1 Hz, 1H), 7.11-7.04 (m, 1H), 7.00-6.98 (m, 1H), 3.86-3.84 (m, 4H), 3.05-3.03 (m, 4H).

S.2.3 (4-(2-Nitrophenyl)piperazin-1-yl)(thiophen-2-yl)methanone (**43**)

Purified by automated flash column chromatography eluting with n-hexane:DCM 50:50 v/v increasing to 0:100 v/v in 10 CV. Obtained in 83% yield as a red solid. ^{1}H-NMR ($CDCl_3$), δ: 7.86 (dd, J_1= 6.5 Hz, J_2= 1.5 Hz, 1H), 7.78 (dd, J_1= 5.1 Hz, J_2= 1.1 Hz, 1H), 7.63-7.59 (m, 1H), 7.46 (dd, J_1= 2.8 Hz, J_2= 1.30 Hz, 1H), 7.40-7.36 (m, 1H), 7.22-7.21 (m, 1H), 7.14-7.12 (m, 1H), 3.78-3.74 (m, 4H), 3.12-3.02 (m, 4H).

S.3 General method for the preparation of aromatic amines 44-49

The corresponding nitro compound **38-43** (1 mmol) was dissolved in EtOH (15 mL/mmol) and added of 10% wet Pd/C (0.1 g/mmol). The suspension was stirred under a H_2 atmosphere at room temperature for 24 hours. The black suspension was diluted with 1:1 DCM/MeOH solution and then filtered through celite under *vacuum*. The filtrate was evaporated at reduced pressure to afford the title compound which was used for the next step without further purification unless otherwise stated.

S.3.1 4-(4-(Thiophen-2-ylsulfonyl)piperazin-1-yl)aniline (**44**)[S1]

Obtained in 79% yield as a light brown solid. ^{1}H-NMR (DMSO-d_6), δ: 8.12 (dd, J_1= 5.3 Hz, J_2= 1.3 Hz, 1H), 7.72 (dd, J_1= 3.7 Hz, J_2= 1.3 Hz, 1H), 7.36 (dd, J_1= 5.3 Hz, J_2= 3.7 Hz, 1H), 6.73- 6.72 (m, 2H), 6.55-6.53 (m, 2H), 4.65 (bs, 2H), 3.11-3.10 (m, 4H), 3.05-3.04 (m, 4H). ^{13}C-NMR (DMSO-d_6), δ: 143.5, 141.9, 134.9, 133.8, 128.9, 126.1, 119.3, 115.1, 50.4, 46.6.

S.3.2 (4-(4-Aminophenyl)piperazin-1-yl)(thiophen-2-yl)methanone (**45**)[S1]

Obtained in 92% yield as a light brown solid. ^{1}H-NMR (DMSO-d_6), δ: 7.82 (dd, J_1= 7.2 Hz, J_2= 1.1 Hz, 1H), 7.49 (dd, J_1= 4.2 Hz, J_2= 1.1 Hz, 1H), 7,19 (dd, J_1= 7.2 Hz, J_2= 4.2 Hz, 1H) 6.78 (d J= 8.9 Hz, 2H), 6.56 (d, J=8.9 Hz, 2H), 4.68 (bs, 2H), 3.82-3.80 (m, 4H), 3.01-2.99 (m, 4H). ^{13}C-NMR (DMSO-d_6), δ: 162.7, 143.3, 142.4, 137.6, 129.9, 129.6, 127.6, 119.2, 115.2, 51.4.

S.3.3 3-(4-(Thiophen-2-ylsulfonyl)piperazin-1-yl)aniline (**46**)

Purified by automated flash column chromatography eluting with *n*-hexane:DCM 40:60 v/v increasing to 0:100 v/v in 10 CV. Obtained in 75% yield as a yellow solid. ^{1}H-NMR ($CDCl_3$), δ: 7.65 (dd, J_1= 3.7 Hz, J_2= 1.3 Hz, 1H), 7.57 (dd, J_1= 2.4 Hz, J_2= 1.3 Hz, 1H), 7.18-7.15 (m, 1H), 7.01-6.95 (m, 2H), 6.80-6.74 (m, 2H), 4.80 (bs, 2H), 3.24-3.20 (m, 4H), 3.01-2.95 (m, 4H).

S.3.4 (4-(3-Aminophenyl)piperazin-1-yl)(thiophen-2-yl)methanone (**47**)

Purified by automated flash column chromatography eluting with *n*-hexane:EtOAC 90:10 v/v increasing to 60:40 v/v in 10 CV. Obtained in 82% yield as a white solid. ^{1}H-NMR ($CDCl_3$), δ: 8.50 (dd, J_1= 3.9 Hz, J_2= 1.1 Hz, 1H),

7.54-7.53 (m, 1H), 7.38-7.36 (m, 1H), 6.72 (dd, J_1= 4.8 Hz, J_2= 1.7 Hz, 1H), 6.70-6.68 (m, 1H), 6.23 (dd, J_1= 5.2 Hz, J_2= 1.1 Hz, 1H), 6.48-6.47 (m, 1H), 4.76 (bs, 2H), 3.29-3.27 (m, 4H), 3.02-2.99 (m, 4H).

S.3.5 2-(4-(Thiophen-2-ylsulfonyl)piperazin-1-yl)aniline (**48**)

Purified by automated flash column chromatography eluting with *n*-hexane:DCM 40:60 v/v increasing to 0:100 v/v in 10 CV. Obtained in 78% yield as a yellow solid. ^{1}H-NMR (DMSO-d_6), δ: 8.09 (dd, J_1= 5.0 Hz, J_2= 1.1 Hz 1H), 7.68 (dd, J_1= 3.8 Hz, J_2= 1.1 Hz 1H), 7.33-7.31 (m, 1H), 6.88 (dd, J_1= 7.8 Hz, J_2= 1.3 Hz, 1H), 6.81-6.78 (m, 1H), 6.64 (dd, J_1= 6.3 Hz, J_2= 1.3 Hz, 1H), 6.53-6.50 (m, 1H), 4.74 (bs, 2H), 3.19-3.14 (m, 4H), 2.92-2.86 (m, 4H).

S.3.6 (4-(2-Aminophenyl)piperazin-1-yl)(thiophen-2-yl)methanone (**49**)

Purified by automated flash column chromatography eluting with *n*-hexane:DCM 50:50 v/v increasing to 0:100 v/v in 5 CV. Obtained in 87% yield as a red solid. ^{1}H-NMR ($CDCl_3$), δ: 7.74 (dd, J_1= 5.1 Hz, J_2= 1.5 Hz, 1H), 7.47-7.42 (m, 1H), 7.40 (dd, J_1= 3.8 Hz, J_2= 1.1 Hz, 1H), 7.25 (dd, J_1= 2.5 Hz, J_2= 1.1 Hz, 1H), 7.11-7.04 (m, 1H), 7.00-6.98 (m, 1H), 4.75 (bs, 2H), 3.86-3.84 (m, 4H), 3.05-3.03 (m, 4H).

Table S.1 Murine 3D-QSAR model data

**Comp.**	**Structure**	**MNV EC_{50}[A] (μM)**	**MNV CC_{50}[A] (μM)**	**Set[B]**	**Activity EC_{50} (μM) Cresset[C]**	**Predicted EC_{50} (μM) Cresset[D]**
	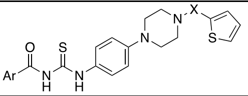					
3	Ar= 4-Me-Ph, X= CO	46.6±0.3	>100	TeS	4.33	4.0
4	Ar= Ph, X= SO_2	44.4±9.5	>100	TrS	4.35	4.30
5	Ar= 4-*t*Bu-Ph, X= SO_2	>100	78.80±27.5	TrS	4.0	4.0
6	Ar= 4-*t*Bu-Ph, X= CO	>100	80.5±33.8	TrS	4.0	4.0
7	Ar= 4-MeO-Ph, X= SO_2	81.1±30.1	>100	TrS	4.09	4.10
8	Ar= 4-MeO-Ph, X= CO	26.1±11.3	>100	Ph, TeS	4.58	4.10
9	Ar= 4-CF_3-Ph, X= SO_2	97.5±4.9	>100	TrS	4.01	4.0
10	Ar= 4-CF_3-Ph, X= CO	75.3±29.1	>100	TrS	4.12	4.10
11	Ar= 4-F-Ph, X= SO_2	>100	59.9±31.4	TeS	4.0	4.0
12	Ar= 4-F-Ph, X= CO	>100	>100	TrS	4.0	4.10
13	Ar= 4-MeOCO-Ph X= SO_2	>100	>100	TrS	4.0	4.0
14	Ar= 4-MeOCO-Ph X= CO	46.6±12.1	>100	TrS	4.33	4.30
15	Ar= 2-Furan, X= SO_2	66.0±35.6	>100	TeS	4.18	4.20
16	Ar= 2-Furan, X= CO	>100	>100	TeS	4.0	4.20
17	Ar= 2-Thiophene X= SO_2	34.7±18.1	>100	TrS	4.46	4.50
18	Ar= 2-Thiophene, X= CO	60.5±29.2	>100	TrS	4.22	4.30
19	Ar= 4-Me-Ph, X= SO_2	>100	49.8±35.6	TrS	4.0	4.0
	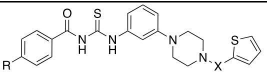					
20	R= Me, X= SO_2	78.2±27.3	>100	TrS	4.11	4.10
21	R= Me, X= CO	92.7±9.2	>100	TrS	4.03	4.0

22	R= H, X= SO_2	99.0±1.7	>100	TrS	4.0	4.0
23	R= H, X= CO	>100	>100	TrS	4.0	4.0
	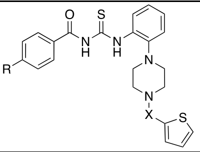					
24	R= Me, X= SO_2	34.0±30.1	>100	TrS	4.47	4.50
25	R= H, X= SO_2	77.5±15.4	>100	TrS	4.11	4.10
26	R= H, X= CO	72.9±46.9	>100	TrS	4.12	4.10
	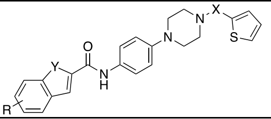					
27	R= H, Y= O, X= SO_2	64.3±41.3	>100	TrS	4.19	4.30
28	R= H, Y= O, X= CO	70.8±30.2	>100	TrS	4.15	4.10
29	R= 5-OMe, Y= O, X= CO	>100	93.2±11.8	TeS	4.0	4.40
30	R= H, Y= NH, X= SO_2	23.7±12.4	>100	Ph, TrS	4.63	4.50
31	R= H, Y= NH, X= CO	25.9±14.2	>100	TrS	4.59	4.60
32	R= 5-Me, Y= NH, X= SO_2	25.8±14.6	>100	Ph, TrS	4.59	4.60
33	R= 5-OMe, Y= NH X= SO_2	25.5±14.3	>100	TrS	4.59	4.60
34	R= 6-OMe, Y= NH X= SO_2	26.2±14.7	>100	Ph, TrS	4.58	4.60
S1	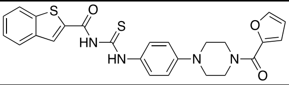	98.4±2.4	>100	TrS	4.01	4.0
S2	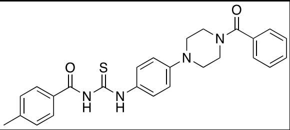	>100	>100	TrS	4.0	4.0
S3	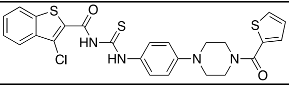	75.0±27	>100	TrS	4.12	4.10
S4	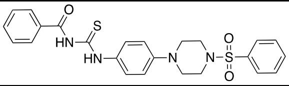	73.2±18.3	71.2±39.6	TrS	4.14	4.20
S5	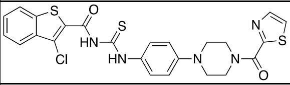	66.1±12.3	>100	TrS	4.18	4.20
S6	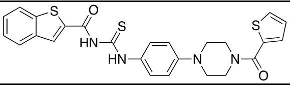	>100	n.d.	TrS	4.0	4.10
S7	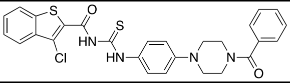	>100	n.d.	TrS	4.0	4.0
S8	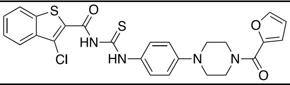	>100	n.d.	TeS	4.0	4.10
S9	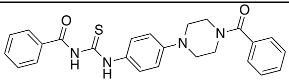	>100	n.d.	TeS	4.0	4.20

[A] The mean values ± standard deviations are shown from at least three independent experiments.
[B] Role of the compounds in the 3D-QSAR model development. Ph: Pharmacophore; TeS: test set; TrS training set.
[C] The experimental activity (EC_{50}) of the data set compounds were converted to its positive-logarithmic scale using the formula: $pEC_{50} = -\log (EC_{50})$ by the Cresset program.
[D] Predicted activity of the 3D-QSAR model.
Compound S1-S9 previously published[S1]

Table S.2 Human 3D-QSAR model data

Comp.	Structure	HuNoV EC_{50}[A] (μM)	HuNoV CC_{50}[A] (μM)	Set[B]	Activity EC_{50} (μM) Cresset[C]	Predicted EC_{50} (μM) Cresset[D]
	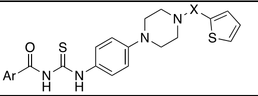					
3	Ar= 4-Me-Ph, X= CO	15.8±13.5	64.5±31.5	TrS	4.80	4.70
4	Ar= Ph, X= SO_2	4.8±5.2	63.9±22.5	Ph, TrS	5.32	5.30
5	Ar= 4-*t*Bu-Ph, X= SO_2	>100	>100	TrS	4.0	4.0
6	Ar= 4-*t*Bu-Ph, X= CO	>100	>100	TrS	4.0	4.0
7	Ar= 4-MeO-Ph, X= SO_2	>100	>100	TrS	4.0	4.10
8	Ar= 4-MeO-Ph, X= CO	39.5±52.4	8.44±3.73	TrS	4.40	4.50
9	Ar= 4-CF_3-Ph, X= SO_2	73.5±46.0	11.21±6.31	TrS	4.13	4.10
10	Ar= 4-CF_3-Ph, X= CO	>100	11.67±9.06	TeS	4.0	4.0
11	Ar= 4-F-Ph, X= SO_2	21.4±9.6	15.26±11.12	TrS	4.67	4.70
12	Ar= 4-F-Ph, X= CO	41.3±50.9	29.53±14.16	TrS	4.38	4.70
13	Ar= 4-MeOCO-Ph X= SO_2	16.4±13.3	>100	TrS	4.79	4.70
14	Ar= 4-MeOCO-Ph X= CO	>100	>100	TrS	4.0	3.90
15	Ar= 2-Furan, X= SO_2	58.7±36.9	63.50±31.81	TeS	4.23	4.30
16	Ar= 2-Furan, X= CO	>100	67.71±24.49	TeS	4.0	5.30
17	Ar= 2-Thiophene X= SO_2	45.4±47.3	17.82±5.00	TeS	4.34	5.1
18	Ar= 2-Thiophene, X= CO	>100	>100	TeS	4.0	4.40
19	Ar= 4-Me-Ph, X= SO_2	71.7±49.1	37.42±11.84	TrS	4.14	4.10
	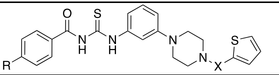					
20	R= Me, X= SO_2	>100	>100	TrS	4.0	4.10
21	R= Me, X= CO	>100	81.3±37.5	TrS	4.0	4.10
22	R= H, X= SO_2	>100	>100	TrS	4.0	4.0
23	R= H, X= CO	>100	68.8±37.5	TrS	4.0	4.0
	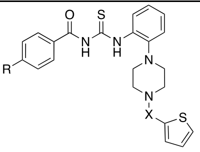					
24	R= Me, X= SO_2	80.4±23.5	>100	TeS	4.09	4.90
25	R= H, X= SO_2	17.4±16.2	>100	TeS	4.76	4.50
26	R= H, X= CO	9.4±4.2	87.5±25.0	TrS	5.19	5.30
	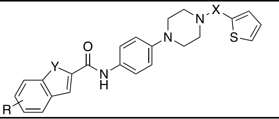					
27	R= H, Y= O, X= SO_2	5.0±1.9	22.0±7.4	Ph, TrS	5.30	5.30
28	R= H, Y= O, X= CO	0.9±0.4	4.1±1.7	Ph, TrS	6.02	5.60
29	R= 5-OMe, Y= O, X= CO	52.5±32.1	87.5±25.0	TrS	4.28	4.20
30	R= H, Y= NH, X= SO_2	15.6±3.3	70.0±21.7	TrS	4.81	5.10
31	R= H, Y= NH, X= CO	6.2±4.4	18.2±9.3	Ph, TrS	5.21	5.30
32	R= 5-Me, Y= NH, X= SO_2	34.9±6.1	>100	TrS	4.46	4.50
33	R= 5-OMe, Y= NH X= SO_2	68.9±37.0	>100	TrS	4.0	4.16
34	R= 6-OMe, Y= NH	60.4±30.2	>100	TrS	4.22	4.10

	X= SO_2					
S1	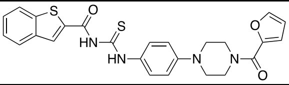	>100	>100	TrS	4.0	4.0
S3	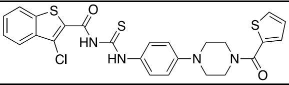	>100	>100	TrS	4.0	3.90
S4	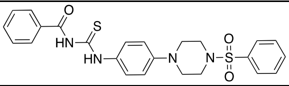	8.4±2.1	>100	TrS	5.08	5.20
S5	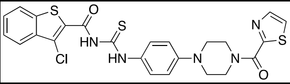	>100	>100	TrS	4.0	4.0

[A] The mean values ± standard deviations are shown from at least three independent experiments.
[B] Role of the compounds in the 3D-QSAR model development. Ph: Pharmacophore; TeS: test set; TrS training set.
[C] The experimental activity (EC_{50}) of the data set compounds were converted to its positive-logarithmic scale using the formula: $pEC_{50} = -\log(EC_{50})$ by the Cresset program.
[D] Predicted activity of the 3D-QSAR model.
Compound S1,S3, S4 and S5 previously published[S1]

References

S1 Giancotti, G.; Rigo, I.; Pasqualetto, G.; Young, M.T.; Neyts, J.; Rocha-Pereira, J.; Brancale, A.; Ferla, S.; Bassetto, M. A new antiviral scaffold for human norovirus identified with computer-aided approaches on the viral polymerase. *Sci. Rep.* **2019**, *9*, 18413.
